# Attitudes towards Future Unemployment and European Cooperation to Reduce Unemployment among 8th Graders in EU/European Countries

**DOI:** 10.3390/ejihpe12020017

**Published:** 2022-02-17

**Authors:** Aleš Trunk, Eva Klemenčič Mirazchiyski, Plamen Vladkov Mirazchiyski

**Affiliations:** 1 International School of Social and Business Studies, Mariborska cesta 7, 3000 Celje, Slovenia; 2 Educational Research Institute, Centre for Applied Epistemology, Gerbičeva 62, 1000 Ljubljana, Slovenia; eva.klemencic@pei.si (E.K.M.); plamen.mirazchiyski@pei.si (P.V.M.)

**Keywords:** civic and citizenship education, future unemployment, future financial wellbeing, job security, employment reinsurance, EU economic cooperation

## Abstract

The focus of this article is on the attitudes among 8th graders in European countries on future unemployment and attitudes towards cooperation among European countries to guarantee high levels of employment and strengthen their economies. This article uses both qualitative and quantitative approaches. For the qualitative approach, a systematic literature review was performed using four databases, starting from 16,873 search results for the 2016–2021 period before systematically limiting them to identify possible predictors used in quantitative analyses. The quantitative part uses secondary analyses of data obtained from 52,788 upper secondary students from 14 EU and one EU associated country from the International Civic and Citizenship Education Study (ICCS) 2016, which is the last available cycle with publicly available data since 2018. The techniques used to analyse the data are descriptive statistics, linear and binary logistic regression, Pearson’s and Spearman’s correlation coefficients, and Principal Component Analysis. This article also considers the theoretical base of the sustainable development definition—it explores youths’ present perceptions of the future in the economic and financial domains.

## 1. Introduction

In the intensive discussions and use of the concept of “sustainable development” since the end 1980s, sustainable development was defined as “*development which meets the needs of the present without compromising the ability of future generations to meet their own needs*” [1] (p. 15). Three core strands of sustainably developed systems have been recognised [2]:

- Economic: has to be able to continuously produce goods and services, maintain viable levels of government and external credit and, finally, avoid extreme sector-related imbalances which damage production in agriculture and industry;
- Social: characterised by fairness in opportunities and distribution, providing social services adequately, which includes education, health, education, gender equity, as well as political participation and accountability;
- Environmental: maintains stable resources, without overexploitation or exhaustion of renewable resources, or spending non-renewable resources where adequate substitutes are available. This involves the maintenance of naturally occurring processes which are not classified as economic resources, like atmospheric stability and biodiversity.

In 2015, the United Nations’ member states adopted the 2030 Agenda for Sustainable Development. The agenda provides a common model to guarantee present and future peace and prosperity for both people and the planet.

“At its heart are the 17 Sustainable Development Goals (SDGs), which are an urgent call for action by all countries […] in a global partnership. They recognise that ending poverty and other deprivations must go hand-in-hand with strategies that improve health and education, reduce inequality, and spur economic growth […]”

[3]

This sustainability model lays out a future where a balance in environmental, societal, and economic aspects is achieved, while improving the quality of life [3]. Due to the interlinked dimensions of sustainable development, there are different definitions of it. Some derive three or four dimensions (as presented above, [3]), other five (e.g., [4]), and some even seven dimensions (e.g., [5]). (To understand sustainability better, terms like “Place”, “Permanence”, and “Persons” can be used. The first term encompasses the three spatial dimensions, the second is the time (fourth dimension), and the last one represents the human dimension. The framework encompassing five dimensions is (arguably) more inclusive and plural, as well as outlining specific sustainability policies [4]. [5] sustains that people stive to meet their needs and aspirations through the economy, community, occupational groups, government, environment, culture, and physiology. These components represent hierarchical levels, and human sustainability can be accomplished by reaching sustainability in all levels.) However, the common framework is actually composed of a set of three main areas or dimensions, namely social, economic, and environmental.

### 1.1. Contribution of the Paper

This article, with a focus on attitudes towards future unemployment and European cooperation to reduce unemployment and strengthen the economies, crosses the social and economic dimensions of sustainable development, based on the model conceptualised and presented in the empirical part of the article. The economic dimension of the model is captured in attitudes towards unemployment, finding a steady job, and possessing a sufficient amount of finances in the future. The social dimension is covered by the future cooperation among the EU countries to reduce unemployment and strengthen the economies, as well as the variables on the home resources of students when responding to specific attitudinal statements.

Another aspect from the definition of sustainable development included in the article is the time dimension, more precisely the present–future scale. Therefore, the special focus of this article is on the attitudes of today’s young people (8th-graders) about the future—the specific focus on unemployment is given more from a community perspective. Specific attitudes to jobs—individual perspective—are also considered. Moreover, there is another element in the discussion of the dimensions of sustainable development: the demand for cooperation.

The right to development is underscored by international solidarity and the duty to cooperate—the effective implementation of all human rights requires international solidarity and cooperation, global partnership for development, policy coherence, coordination, and integrated approaches at all levels. The aspiration to make the right to development a reality for all also informs the contemporary global development policy framework—the 2030 Agenda SDGs. The Agenda reaffirms that:

“We are determined to mobilise the means required to implement this Agenda through a revitalised Global Partnership for Sustainable Development, based on a spirit of strengthened global solidarity, focused in particular on the needs of the poorest and most vulnerable and with the participation of all countries, all stakeholders and all people.”

[6]

Different political and social events brought the concerns related with the future feasibility of cooperation and integration activities in Europe. Some of the most pressing issues in 2016/2017 in Europe were related with migrants and refugees, (unemployment, public finances and inflation), as well as the issues of foreign and security policies [7,8].

The fight against terrorism and unemployment, as well as the protection of the environment, on the EU average, were the policy areas that more than three quarters of respondents pointed out as being in need of intervention by the EU, as reported by the Eurobarometer from April 2018 [9,10]. (Please note that the participants in the Eurobarometer survey are older than the ones included in our secondary analyses.) The change when comparing the results from Eurobarometers 2016 and 2018 [10,11], however, is important in determining how respondents perceive the current actions the EU took in these areas. In 2018, 32% of the respondents see the EU’s fight against terrorism as adequate (a significant increase of 9% since 2016). Similarly, 29% of the respondents in 2018 have the same view on the measures to reduce unemployment (a significant increase of 6% since 2016), while the share of those who see the unemployment measures as insufficient dropped by 10% (69% in 2016, 59% in 2018) [9].

Throughout the history, the concept of solidarity has been institutionalised with the intent to prevent future risks and adversities (e.g., social marginalisation, unemployment, illness, and even natural disasters). However, evidence from recent history shows the opposite effects [12]—a decrease in solidarity and, at the same time, populist, nationalist, and anti-establishment political parties rose in several European countries. These factors brought complicated and perplexing dynamics to the original European integration idea, but not only that—they also added more complexity to the cross-country economic and political relations [13]. Furthermore, the debate in recent years has faced the questions on the need for stronger cooperation across Europe, as well as questions on the effects of the financial crisis on the economies [7].

From an economic perspective, the literature and (EU) political agenda on unemployment could be perceived as a topic of future cooperation and solidarity among the EU countries. In the case of the EU unemployment reinsurance model, this could be perceived, in its economic and social consequences, as crossing both the economic and the social dimension of sustainable development. The European Commission published its new work programme at the beginning of 2020. “An Economy that Works for People” is the third priority in the programme where an intended proposal for a European Unemployment Reinsurance Scheme was placed. The topic has been discussed since 2012, as a consequence of the 2008 financial and economic crisis. The idea, however, had already appeared in the 1970s when the debate on the Monetary Union started, and the idea of a fiscal capacity for the Eurozone was analysed as an instrument of stabilisation and redistribution. Most recently, the annual report on employment and social aspect of the European Semester, Parliament’s Employment and Social Affairs Committee welcomed the Commission’s continued intention to design a European Unemployment Benefit Reinsurance Scheme [14].

“We must also do more to support those who lose their jobs because of external events that affect our economy. This is why I will propose a European Unemployment Benefit Reinsurance Scheme. This will protect our citizens and reduce the pressure on public finances during external shocks.”

[15] (p. 10)

The proposal was made by the new President of the European Commission. It reiterated an idea which became popular among most researchers, who insisted on strengthening Europe’s social dimension [16].

A study in 13 EU countries with a total of 19,500 respondents found that just about 3% said they disagree with any kind of unemployment risk sharing. On the contrary, around 6% supported all forms of these schemes. The support depends on the design features, as well as on respondents’ attitudes and background characteristics [17]. Individual attitudes on European integration, a cross-border dimension, have influenced the level of administration. The results on between-country redistribution are somewhat similar, although less clear [17].

To cover the present–future scale of the sustainable development attitudes of today’s young generations, who are the future adult generation, seems to be the proper approach. The purpose of the European student questionnaire ICCS 2016 was to assess different aspects of civic and citizenship education, relevant to the European context, as well as the social and the important political settings with high importance in this region. In recent years, different social and political circumstances brought concerns related with the future of cooperation and integration across Europe. Some of the most pressing issues Europe faced in 2016/2017 were related to migrants and refugees, as well as the economy—inflation, raise of unemployment, and public finances—and foreign and security policies [7,8].

As this article is focused on European and additional topics related to attitudes on unemployment and European cooperation, as well as the views of the European students surveyed regarding their individual financial and job-related future, the dataset from the European student questionnaire, as well as the international ICCS student questionnaire will be used for secondary analyses. However, to identify possible predictor variables from the International ICCS student questionnaire for the analyses, a systematic literature review of articles and books in the publishers’ databases was performed. At first, a total of 16,873 published items was identified. This theoretical approach then led to the empirical one, where the datasets from several European countries, with a total of 52,788 students at grade 8 (grade 9 in Norway), were used.

The findings show that, in general, 8th grade students’ optimism about their job-related future and financial wellbeing across European countries is around the European average. They are also supportive of European cooperation towards both guaranteeing high levels of employment and strengthening the economies. Gender has shown a limited association with students’ opinion on their future financial wellbeing and European cooperation to strengthen the economies. Gender is, however, strongly related with students’ perception of the rise of unemployment and the fact that the economy will be weaker. Family SES and migration status show an association with students’ opinion on their future financial wellbeing in just one country. However, in some countries SES is related with the students’ belief that European cooperation will strengthen the economies and that the economies will be weaker. As for the students’ anticipation of the rise of poverty and unemployment, SES shows a significant relationship in nearly half of the countries. The endorsement of EU cooperation is equally related with students’ perception that the economies will be weaker and that there will be a rise in unemployment in Europe in some countries. The immigration status and expected further education show a limited relationship with all constructs. Student achievement in civic and citizenship education is the variable which shows the strongest relationship with all dependent variables, in nearly all countries.

### 1.2. Approach to Identify Predictors: Systematic Literature Review

An important part of this study is to identify the relevant predictors of the outcome variables (“The economy will be weaker in all European countries”, “There will be a rise in poverty and unemployment in Europe”, “European cooperation to guarantee high levels of employment”, “European countries should cooperate to strengthen their economies”). The identification of these variables will help construct the statistical models. The identification of the predictors was done through a systematic literature review on the sources relevant to the topic. For details on the systematic literature reviews, please refer to the Supplementary Material.

The systematic literature review of the relevant sources (see the Supplementary Material for details) is very important for the purpose of this article because it helps identify relevant predictors for the secondary analysis. Although the literature did not always show a direct association with attitudes, it provided important statistical findings. Moreover, statistics outside Europe were taken into consideration, and the target population was broader than 8th graders. Several predictors for our secondary analyses were identified: immigration status (e.g., [18,19]), gender (e.g., [19,20,21,22]), job insecurity/satisfaction (e.g., [23,24]), and levels of educational attainment (e.g., [21,22,25,26,27,28]). These variables are the outcome of the systematic literature review, and the ones related to them from the ICCS database will be used in the statistical analyses.

Findings from both the 2009 and 2016 cycles of ICCS show that attitudes are often associated with the family’s socio-economic status (SES) and civic knowledge [29,30,31,32]. Therefore, these two variables were also considered in the analysis.

The empirical part of this paper, presented below, includes several analyses on different attitudes towards personal finance in the future, including those related to satisfaction with future jobs and attitudes towards future scenarios—future unemployment and cooperation among European countries to reduce unemployment, taking into consideration the identified predictors listed above.

## 2. Materials and Methods

### 2.1. Sample Description and Source for Secondary Analyses

The 2016 cycle of the International Civic and Citizenship Education study (ICCS 2016), internationally coordinated by the International Association for the Evaluation of Educational Achievement (IEA), and nationally by the national study centres, was assessed at the 8th grade of schooling, where the average student age is no less than 13.5. In the 2016 cycle, 24 educational systems were part of the study. This study uses only data from the EU countries: Belgium (Flemish) (*N* = 2931), Bulgaria (*N* = 2966), Croatia (*N* = 3896), Denmark (*N* = 6254), Estonia (*N* = 2857), Finland (*N* = 3173), Germany (North Rhine-Westphalia) (*N* = 1451), Italy (*N* = 3450), Latvia (*N* = 3224), Lithuania (*N* = 3631), Malta (*N* = 3764), Netherlands (*N* = 2812), Slovenia (*N* = 2844), and Sweden (*N* = 3264). The data from Norway, an EU-associated country, is also included (*N* = 6271), because of its membership of the European Economic Area (EEA). The total number of students in this study is 52,788, all of them 8th graders, with the exception of students from Norway. Norway participated with 9th-grade students [33]. The data collections were conducted in 2016, the first international results were reported at the end of 2017, and the international database was released in March 2018.

### 2.2. Data and Description of Variables, including Descriptive Statistics

The data used in this study come from two ICCS 2016 instruments. The basic descriptive statistics and the later secondary analyses use the ICCS 2016 European regional questionnaire data. The data on students’ background characteristics are from the ICCS 2016 International Student Questionnaire. The description of the variables from the International European Regional Module and the analysis plan are given in Table 1. The actual questions can be found in the international version of the questionnaire in the ICCS User Guide [34]. The ICCS is publicly available online [35].

The ICCS has complex sampling and assessment designs. In each country, a multistage cluster sample with a probability proportional to the size of the schools was drawn. The testing component of ICCS uses a multi-matrix sampling of items, which results in five imputed scores for each student, known as “plausible values” (PVs). For more details on how PVs are produced in ICCS 2016, see the ICCS 2016’s technical report [33]. For a more general methodological overview of PVs in large-scale assessments and the imputation model, please refer to [36]. All analyses in this article were performed using the R Analyzer for Large-Scale assessments (RALSA) [37], which can handle all complex sampling and assessment design issues related with the analysis of large-scale assessment data.

The tables with the basic descriptive statistics on the variables can be found in the Supplementary Material (Tables S1–S4). Here, only a brief summary is presented. The descriptive analyses show that 8th graders (9th graders in the case of Norway) in ICCS 2016 have mostly positive views on their future relating to job and finances. The variation on these statements is rather small across countries. The majority of students think they would very likely or likely find a steady job (95.2% on average), find a job they would like (91.5% on average), and earn enough money to start a family (95.6% on average). However, lower percentages (78%) and more variation across countries on students’ anticipation of their future financial situation in comparison to that of their parents were found. In Belgium (Flemish) and Sweden, student reports are more than 10% below the European ICCS 2016 average.

The descriptive statistics also show that, on average, 42.9% of the students are of the opinion that the economy would weaken in all European countries, and 52.4% foresaw a rise in poverty and unemployment in Europe in the next 10 years, but Italy and Slovenia have around 10% more students who find this more likely than the European ICCS average for this statement. From all countries taking part in this study, Denmark had the lowest percentages of students agreeing with these statements, which is more than 12% below the European ICCS 2016 average.

Nearly all of the students favoured the cooperation among European countries to guarantee high levels of employment (94.8%) and to strengthen their economies (94.3%).

## 3. Results

### 3.1. Students’ Perception of Their Individual Future Related to Employment

**Figure 1:**
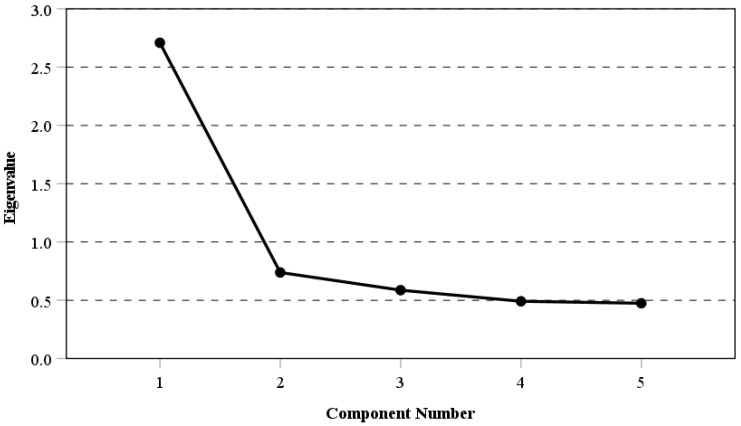
Scree plot from the PCA for the “Financial wellbeing expectations for the future” scale.

To be able to analyse the relationship between students’ future financial wellbeing expectations, a continuous scale was constructed using four-category variables (see Table 1). Principal Component Analysis (PCA) was used to construct the scale and test its dimensionality, applying the total student weight. First, the original four-category variables were recoded, so that their categories are in increasing order of their magnitude (“Very unlikely”, “Unlikely”, “Likely”, and “Very likely). The recoded variables were then used to test the dimensionality of the scale and the individual contribution of each variable assuming a single-factor structure. The single-factor structure was confirmed by the results in Table 2. A total of five possible factors were identified. The first one has the highest eigenvalue (2.71), which is more than 3.5 times higher than the next component and explains 54.2% of the explained variance. Thus, a single factor solution was found. These results are confirmed by the scree plot in Figure 1. The reliability (Cronbach alpha) of the scale is 0.783, which is satisfactory, bearing in mind that all statistics will be computed on the group level and no decisions for individuals will be made.

Individual scores were produced for each student record to make the new scale. The original metric of the scale was, with a mean of 0 and a standard deviation of 1. It was altered to be with a mean of 50 and a standard deviation of 10 to put it on the same metric as the other scales in ICCS 2016 and to ease the interpretation of the results. The country averages on the new scale are presented in Table 3, below. As the table shows, there is little variation in the averages on the scale across the education systems. The averages are the highest in Denmark (53.44 points), Germany, North-Rhine Westphalia (53.30 points), and The Netherlands (53.20 points). They are lowest in Bulgaria (48.11 points) and Croatia (48.65 points). The average across all education systems is 51.16 points.

The scale was used in a multiple regression analysis, where it was added as a dependent variable, and the predictor variables were added as independent ones: gender, immigration background, students’ future expected educational attainment (ISCED); students’ family SES; students’ civic knowledge scores; and students’ endorsements of the EU cooperation scale. Students’ gender was added as a dummy-coded variable with the category for the male students as the reference category. This was done to test the significance of the differences between boys and girls. All other variables were added unaltered, except for the students’ immigration status, which was reverse-coded, so that higher values represent less migration background. None of the variables is significantly related with the scale, and the coefficients are rather weak. In a separate analysis, only the students’ gender was entered as a single predictor of the scale with dummy coding, where the boys are the reference category to test the differences by gender without any other predictors. Significant differences by gender were found only in Lithuania, where girls are nearly one point lower than boys in their expected future financial wellbeing. The rest of the individual variables were tested for the strength and significance of their relationship with the “Financial wellbeing expectations for the future” scale using correlations. The students’ immigration background and the students’ expected educational attainment are ordinal variables and, thus, a Spearman rank correlation was used. On the other hand, the students’ SES, students’ civic knowledge (five PVs), and the students’ endorsement of EU cooperation are continuous variables, and a Pearson correlation was used. The Pearson correlation between the “Financial wellbeing expectations for the future” scale and students’ civic knowledge shows a weak correlation in nine countries with coefficients from −0.02 to 0.02. In some of the education systems (Belgium [Flemish], Denmark, Lithuania, and Malta) these coefficients are even negative (i.e., the higher the knowledge, the less the expectations tend to be), but the coefficients are very close to zero. In six education systems (Croatia, Estonia, Italy, Latvia, the Netherlands, and Sweden), the correlation coefficients are strictly equal to zero.

The coefficients from the Spearman correlation between the “Financial wellbeing expectations for the future” scale and students’ immigration status are close to zero in all educational systems. In all education systems the correlation coefficients are insignificant, with Norway being the only exception (*p* < 0.05). However, even in this case the correlation is quite weak (r = 0.03).

The Spearman correlation coefficients between the “Financial wellbeing expectations for the future” scale and students’ expected further education vary between −0.03 and 0.03, i.e., it is very close to zero. In Belgium (Flemish), Malta, and Norway the coefficients are exactly zero (no correlation between the variables). None of the correlation coefficients across all education systems are statistically significant (*p* > 0.05).

The Pearson correlation coefficients between the “Financial wellbeing expectations for the future” scale and the students’ family SES vary from −0.02 to 0.04. The strongest correlation is found in Norway (0.04), and it is the only one statistically significant (*p* < 0.05). However, it is still very weak and close to zero. The correlation coefficients in the rest of the education systems are also close to zero, and in Croatia and Italy they are exactly zero.

The Pearson correlation coefficients between the “Financial wellbeing expectations for the future” scale and students’ endorsement of EU cooperation in all education systems are around zero, ranging from −0.03 to 0.03, and in Croatia, Finland, and Slovenia they are exactly zero, i.e., there is no association between the variables. The coefficients in all education systems are statistically insignificant.

### 3.2. Students’ Attitudes towards Future Unemployment in Europe

The two analyses presented here use the students’ opinion on (1) the economy will be weaker in all European countries; and (2) there will be a rise in poverty and unemployment in Europe. These outcome variables were dichotomised (see Table 1), as 0 (unlikely or very unlikely) and 1 (likely or very likely), and used as dependent variables in binary logistic regression. The binary logistic regression predicts whether students find it likely or very likely that the economy will become weaker, and if there will be a rise in unemployment and poverty. The purpose of these two analyses is to identify the best predictors of students’ opinion on whether these events are likely to happen. Both analyses use several predictor variables (see Table 1).

The first analysis uses the dichotomised variable “The economy will be weaker in all European countries”, where the categories are “Very unlikely or unlikely” and “Very likely or likely”. The results are presented in Table S5 in the Supplementary Material. The interpretation of the regression coefficients for each single predictor needs to consider that it is controlled for by all other predictors in the model. Students’ gender is a significant predictor of the likelihood of the students’ opinion that the economy will be weaker in Belgium, Italy, Malta, Norway, and Slovenia (*p* < 0.05). The coefficients are computed, as the gender variable has been added in the model as dummy-coded, with boys as the reference category. The coefficients for this variable show the difference of girls compared to boys and are all positive, meaning that, in general, girls are more likely to find that the economy will be weaker in all European countries.

The students’ immigration status is a significant predictor of the likelihood that the economy will be weaker (according to the students) only in Latvia (*p* < 0.05). The coefficient is negative, i.e., Latvian students who have less immigration background tend to find it more likely that the economy in all European countries will be weaker, and vice versa: students with more immigration background tend to be more optimistic.

Students’ endorsement of EU cooperation is a significant predictor of the likelihood that the economy will be weaker (according to the students) in Bulgaria, Italy, Norway, and Slovenia (*p* < 0.01). The coefficients on EU cooperation in these countries are positive, which means that the more students endorse the EU cooperation, the more they tend to find it likely that the economy will become weaker.

The students’ expected further education is a significant predictor of the likelihood that the economy will be weaker only in Estonia (*p* < 0.05), where it is negative. That is, Estonian students who expect to go further in their education also tend to find it unlikely that the economy in all European countries will become weaker.

The students’ family SES is significantly related with how much students think it is likely that the economy in all European countries will become weaker in three countries: Croatia, Italy, and Malta. The coefficients are negative, which means that the higher the family SES, the less the students tend to find it likely that the economy in the European countries will become weaker.

Civic knowledge is the strongest predictor of how much students find it likely that the economy will be weaker in European countries. The coefficients are statistically significant in all European countries and are negative, meaning that the higher the students’ civic knowledge, the less likely it is that they think the European economies will be weaker.

The second binary logistic regression analysis uses the dichotomised statement “There will be a rise in poverty and unemployment in Europe”, where the categories are “Very unlikely or unlikely” and “Very likely or likely”. The predictors are the same (see Table 1). The results are presented in Table S6 in the Supplementary Material.

The students’ endorsement of EU cooperation shows a significant relationship with the opinion of students that there will be poverty and unemployment in Europe (dependent variable), Germany (North-Rhine Westphalia), Italy, Lithuania, and Malta (*p* < 0.05). The relationship in Germany (North-Rhine Westphalia), Italy, and Malta is positive, which means that the more students endorse EU cooperation, the more they tend to think that it is likely that there will be a rise in poverty and unemployment. On the contrary, in Lithuania the coefficient is negative, meaning that the more the Estonian students endorse EU cooperation, the less they tend to think it is likely that there will be poverty in Europe.

Students’ gender is significantly related with the outcome variable (“There will be a rise in poverty and unemployment in Europe”) in almost all education systems, except for Belgium (Flemish), Denmark, Germany (North-Rhine Westphalia), the Netherlands, and Sweden. The variable is added to the model as a dummy-coded variable and shows the difference between boys and girls, with boys being the reference category. The coefficients are positive, meaning that girls tend to be more pessimistic, i.e., they tend to think that it is more likely that there will be poverty and unemployment in Europe.

The students’ immigration status is positively and significantly related with students’ opinion on the likelihood of more poverty and unemployment in Europe in Croatia, Latvia, and Slovenia. The relationship in Croatia and Slovenia is positive, i.e., the less immigrant background students have, the more probable it is that they will find it likely there will be more poverty and unemployment in Europe. On the other hand, the relationship in Latvia is negative, meaning that the less migrant background the students have, the less probable it is that they will find it likely there will be poverty and unemployment in Europe.

The expected further education is significantly related with the students’ opinion on the rise of poverty and unemployment in Europe in Denmark only (*p* < 0.05) where it is negative, i.e., the further the students plan to go in their education, the more unlikely they tend to find that there will be a rise in poverty and unemployment in Europe.

The family SES is negatively and significantly related with students’ opinion on the probability that there will be a rise of poverty and unemployment in Europe in nine countries: Bulgaria, Italy, Malta, the Netherlands, Slovenia, and Sweden (*p* < 0.05). The negative relationship in these countries shows that as the family SES increases, the probability that students find it likely that there will be a rise in poverty and unemployment in Europe tends to decrease.

Civic knowledge is related with students’ opinion on the rise of poverty and unemployment in EU in all countries but Belgium (Flemish), Bulgaria, Estonia, Finland, and Slovenia. In all other countries the relationship is statistically significant (*p* < 0.05) and negative, meaning that the higher the civic knowledge of students, the less likely it is that they think there will be a rise in poverty and unemployment in the EU.

### 3.3. Students’ Attitudes towards European Cooperation to Reduce Unemployment and Strengthen the Economy

The two analyses presented here use the students’ opinion on (1) European countries should cooperate to guarantee high levels of employment; and (2) European countries should cooperate to strengthen their economies. These variables were dichotomised (see Table 1), as 0 (“Disagree” and “Strongly disagree”) and 1 (“Agree” and “Strongly agree”), and used as dependent variables in binary logistic regression. The purpose of these analyses is to identify the best predictors which can predict whether students agree with these statements (“European countries should cooperate to guarantee high levels of employment, and strengthen their economies”). Both analyses use the following variables as predictors: gender, immigration background, students’ future expected educational attainment (ISCED); students’ family SES; and civic knowledge.

The “Students’ endorsement on European cooperation” scale was excluded from these analyses because the same items used as dependent variables were also used to produce this scale and would add collinearity to the models.

The first analysis uses the dichotomised variable “European countries should cooperate to guarantee high levels of employment”, where the categories are “Strongly disagree or disagree” and “Strongly agree or agree”. The binary logistic regression model was computed after normalising the weights because of oversampling in Germany (North-Rhine Westphalia), Italy, and the Netherlands, which resulted in excessively large sampling weights in some cases and in a perfect separation of the model. The results are presented in Table S7 in the Supplementary Material.

Students’ gender is positively related with the probability of students agreeing that European countries should cooperate to guarantee high levels of employment. Students’ gender is entered in the model as a dummy-coded variable, with boys being the reference category, so the coefficients represent the difference for girls. The positive statistically significant coefficients were found only in Malta and Norway, meaning that in these two education systems girls are more likely to agree that European countries should cooperate to guarantee high levels of employment.

The immigration status of the students is positively and significantly related with the students’ opinion on European cooperation to guarantee high levels of employment in six countries—Bulgaria, Croatia, Denmark, Estonia, Italy, and Latvia (*p* ≤ 0.05). The positive coefficients in these countries mean that students with less migration background are more likely to agree.

The students’ expected further education is not related with the students’ opinion on European cooperation to guarantee high levels of employment only in Bulgaria, Croatia, and Germany (North-Rhine Westphalia). In all other countries the relationship is positive and statistically significant (*p* ≤ 0.01). This means that the more students expect to go further in their education, the more likely it is that they will agree that there should be European cooperation to guarantee high levels of employment.

Students’ SES is positively and significantly (*p* < 0.05) related with students’ opinion on European cooperation to guarantee high levels of employment in seven education systems: Bulgaria, Finland, Germany (North-Rhine Westphalia), Italy, Malta, the Netherlands, and Norway. This means that in these education systems higher-SES students are more likely to agree on European cooperation to guarantee high levels of employment.

The second analysis uses the dichotomised variable “European countries should cooperate to strengthen their economies”, where the original categories were recoded as “Strongly disagree or disagree” (0) and “Strongly agree or agree” (1). The binary logistic regression model was computed after normalising the weights because of oversampling in Germany (North-Rhine Westphalia), Italy, and the Netherlands, which resulted in excessively large sampling weights in some cases and in a perfect separation of the model. The results are presented in Table S8 in the Supplementary Material.

Students’ gender is positively and significantly related with the probability of students agreeing that European countries should cooperate to strengthen their economies only in Bulgaria, meaning that Bulgarian girls are more likely to agree that European countries should cooperate to strengthen their economies.

The immigration status of the students is significantly related with the students’ opinion that European countries should cooperate to strengthen their economies in Bulgaria, Malta, and Norway (*p* < 0.05). The positive coefficients in Bulgaria and Malta mean that students with less migration background in these countries tend to agree. On the other hand, the coefficient is significant and negative in Norway, which means that students with less migration background are likely to disagree that European countries should cooperate to strengthen their economies.

The students’ expected further education is not related with the students’ opinion that European countries should cooperate to strengthen their economies in all countries but Germany (North-Rhine Westphalia) and Lithuania. The relationship in Germany (North-Rhine Westphalia) is negative and statistically significant (*p* ≤ 0.05), meaning that the higher students tend to go in their education, the more they tend to disagree. In Lithuania, where a positive and statistically significant relationship is found, as the higher students expect to go further in their education, the more likely it is that they will agree that there should be some European cooperation to guarantee high levels of employment.

Students’ SES is negatively and significantly (*p* < 0.05) related with the students’ opinion that European countries should cooperate to strengthen their economies only in Belgium (Flemish), Croatia, and Slovenia. This means that in these education systems the higher-SES students are less likely to agree that European countries should cooperate to strengthen their economies.

## 4. Discussion

The descriptive analyses in the first part of this study show that, in general, the surveyed students in European countries participating in the ICCS 2016 cycle expressed optimism about their respective futures, as well as about the cooperation among European countries to reduce unemployment. The majority of students across countries felt confident that they would find a steady job, find a job they liked, and earn enough money to start a family. Our further analyses were based on predictors identified through a systematic literature review which identified several possible predictors: immigration status, gender, job insecurity, levels of educational attainment. The SES was also included as a predictor in the models, as this indicator provides the only available information of socio-economic information (see the limitations below).

Vasilopoulou and Talving [38] argue that the macroeconomic context is an essential predictor of attitudes towards transnational financial assistance, but has been omitted from previous studies. Data from the 2014 European Election Studies (EES) Voter Study conducted in 28 EU countries demonstrated that citizens residing in poorer EU countries show less support for fiscal solidarity than the ones living in more affluent countries. The heuristic function of a country’s affluence moderates the relationship between individual-level utility and identity considerations, on the one hand, and the willingness for solidarity with member states with economic hardship, on the other. If the economic situation in a country is below optimal, citizens have negative views on providing help to others. This attitude remains regardless of the individual-level utilitarian and identity considerations [38]. A recent study by Franchino and Segatti [39] finds that individuals with high-income and right-wing inclinations who also have a weak European identity and view EU membership negatively are also the ones that will most likely stand against the European fiscal union. Our analyses tried to follow this pattern—to focus on individual-level predictors. However, our results have shown that the SES and the migration status are mostly unrelated to the outcome variables. At the same time, it needs to be acknowledged that the analyses use data from eight-grade students and not adults. Thus, the results from the analyses are only pertinent to eight-grade students, which in most countries are at the age of approximately 14 years.

The “Financial wellbeing expectations for the future” scale constructed for the purpose of this article shows that in most education systems the average scores are at or above the centre point of the scale (50 points) and the grand average is also above it (51.16 points). The only exceptions are Bulgaria, Croatia, and Belgium (Flemish), which are below the centre point and the grand mean. Bulgaria and Croatia are not the only two post-communist European countries in this study (Estonia, Lithuania, Latvia, and Slovenia are post-communist countries as well), but are the ones who have joined the EU last and are at a lower stage of their economic development compared to the others. Some of the predictors of the scale in this study did show a relationship with the scale in some countries. Gender has shown a relationship only in Lithuania, where girls are more pessimistic. The students’ migration status and the family SES show significant relationship only in Norway, where the students with less immigration status and those of higher-SES families tend to have higher expectations on their future financial wellbeing, although the relationship in both cases is rather weak.

The binary logistic regression analysis for the students’ expectations that the economy of European countries will be weaker revealed some interesting results. In Belgium (Flemish), Italy, Malta, Norway, and Slovenia, female students are more likely to have the opinion that the economy in European countries will be weaker. In Latvia, students with less immigration background tend to find it more likely that the economy in all European countries will be weaker, and vice versa: students with more immigration background tend to be more optimistic. Students’ endorsement of EU cooperation is a significant predictor of the likelihood that the economy will be weaker only in Bulgaria, Italy, Norway, and Slovenia—the more students endorse EU cooperation, the more they tend to find it likely that the economy will become weaker. In just one of 15 the European countries (Estonia) is students’ expected further education a negative and significant predictor of the likelihood that the economy in European countries will be weaker—students who expect to go further in their education also tend to be more optimistic. The students’ family SES is a significant predictor in just three countries (Croatia, Italy, and Malta), all of them in South Europe: the higher the family SES, the more the students tend to find it unlikely that the economy in the European countries will become weaker. Civic knowledge appears to be the best predictor of students’ expectations that the economy will be weaker in all the countries in this analysis. The higher the civic knowledge, the less likely it is that students will think the economy will be weaker. In some countries the regression coefficients are quite high—in Malta, it is more than 10 score points.

The binary logistic regression analysis with the students’ expectations about the rise in poverty and unemployment in Europe shows a relationship with students’ endorsement of EU cooperation only in Germany (North-Rhine Westphalia), Italy, Lithuania, and Malta. The students’ opinion is, however, more related with students’ gender and civic knowledge—in 10 countries girls appear to be more pessimistic than boys, and higher achievers tend to be more optimistic than lower achievers. The results on gender do not stand mostly in high-income countries—Belgium (Flemish), Denmark, Germany (North-Rhine Westphalia), the Netherlands, and Sweden. The results on civic knowledge, on the other hand, are mixed—no significant relationship was found in Belgium (Flemish), Bulgaria, Estonia, Finland, and Slovenia. That is, the high- and low-income pattern does not show a definitive pattern. The immigration status is related with the opinion that there will be more poverty and unemployment in Europe in three post-communist countries—Croatia, Latvia, and Slovenia. In Croatia and Slovenia, the students with less immigration status are more pessimistic, while in Latvia they are more optimistic. This result is a bit surprising because in 2016 (the year when the ICCS data were collected) Croatia and Slovenia had a much higher gross domestic product (GDP) than Latvia [40]. The expected further education is related with the expected rise in unemployment and poverty in Europe only in Denmark, where those who expect to go further in their education find it more unlikely that the rise will happen. As for the SES, in six countries students from higher-SES families find it more unlikely that the rise in unemployment and poverty will happen. However, no distinct pattern across countries can be found.

Nearly all of the students in ICCS 2016 favoured the cooperation among European countries to guarantee high levels of employment and to strengthen their economies. As for how the future in Europe would look like, on average, 43% of students anticipate that the economy would weaken in all European countries. Moreover, 52% foresaw a rise in poverty and unemployment in Europe. Students in Denmark, however, are the most optimistic in these regards, with the lowest percentages of agreement. The binary logistic regression analyses for cooperation on both fighting unemployment and strengthening the European economy show a relationship with students’ gender in just one country (Denmark), where girls are more likely to disagree that there shall be cooperation. The immigration status is not significantly related with the students’ opinion on European cooperation for guaranteeing high levels of employment. The immigration status is, however, a significant predictor of the students’ opinion on European cooperation to strengthen the economy in three countries. In two of these cases (Bulgaria and Malta) this relationship was found positive, i.e., students with less of a migrant background tend to agree. In Norway, the relationship is negative, i.e., students with less migration background are more likely to disagree. The expected further education is related with students’ opinion on European cooperation for both guaranteeing high levels of employment and strengthening economies in just two education systems (Estonia and Sweden), where students with expected higher further education are likely to agree with cooperation for both high employment and strengthening economies. For the European cooperation aimed at strengthening the economies, the results are similar: Germany (North-Rhine Westphalia) and Lithuania are just two of the countries where a significant relationship was found. However, while in Germany (North-Rhine Westphalia) the relationship is negative (i.e., students who plan to obtain further higher education are more likely to disagree), in Lithuania the relationship is positive (i.e., students who plan to go further in higher education tend to agree). As for the students’ family SES, it is not significantly related with students’ opinions on European cooperation to guarantee high levels of employment, but it is significantly related with European cooperation to strengthen the economies in Belgium, Croatia, and Slovenia.

Students’ civic knowledge is significantly related with students’ opinion on European cooperation for both guaranteeing high levels of employment and strengthening the economies in most countries. The only exception for the levels of employment is Germany (North-Rhine Westphalia), and for strengthening the economies—Malta, Norway, and Sweden.

## 5. Conclusions

The main purpose of this article was to identify the strongest predictors of (1) students’ perception of their individual future related to employment; (2) students’ attitudes towards future unemployment in Europe; (3) students’ attitudes towards European cooperation to guarantee high levels of employment; and (4) students’ attitudes towards European cooperation to strengthen the economy. Based on the results of these analyses, it can be said that:

1. Gender has shown a limited association with students’ opinion on their future financial wellbeing and European cooperation to strengthen the economies (one country only). However, gender is strongly related with students’ perception that unemployment will rise (10 countries) and that the economy will be weaker. The rest of the background student characteristics have shown no relationship with students’ opinion on their own future financial wellbeing.
2. Family SES and migration status show an association with students’ opinion on their future financial wellbeing in just one country. As for the students’ anticipation of the rise in poverty and unemployment, SES shows a significant relationship in six countries. In all countries, SES is unrelated with students’ opinion on European cooperation to guarantee high levels of employment. However, in three countries it is related with the students’ opinion on European cooperation to strengthen the economies and that the economies will be weaker. The relationship between SES and the cooperation between countries to strengthen their economies is, however, negative—students with high SES tend to disagree on such cooperation. This is quite surprising by itself, but at the moment no explanation can be provided.
3. The endorsement of EU cooperation is equally related with students’ perception that economies will be weaker and that there will be a rise in unemployment in Europe (four countries).
4. The immigration status and expected further education show a limited relationship with all constructs. The highest number of countries showing a relationship with the dependent variable concerns the students’ opinion on the rise of unemployment in Europe.
5. Students’ achievement in civic and citizenship education is the variable which shows the strongest relationship with all dependent variables. The highest number of countries where the association was found concerns the students’ opinion that the economies will be weaker (all 15 countries) and the European cooperation to guarantee high levels of employment (14 countries). As for the other two dependent variables, the rise of unemployment and the European cooperation to strengthen economies, the relationship was found in most countries (12 and 10, respectively).

Two of the aspects analysed in this article, the attitudes toward European cooperation to guarantee high levels of employment and to strengthen their economies, are related with the frequently appearing concept of solidarity across EU member countries. The different crises in Europe (financial, including risks of unemployment, migration crises, Brexit, etc.) in the last 15 years or so often bring different aspects to the discussion on solidarity. Our article is focused on financial and welfare (social) solidarity, by investigating the attitudes of the youths (8th graders) towards their own future (financial stability), the future of Europe, and the importance of cooperation among European countries to reduce unemployment and strengthen the economy. Gerhards, Lengfeld, Ignácz, Kley, and Priem [41] argue that European solidarity exists if four criteria are met: (1) the majority of all Europeans support the European solidarity idea; (2) the EU constitutes a specific space of solidarity, which is distinct from both global and national solidarity; (3) EU citizens are prepared to sacrifice resources for European solidarity; and (4) social and political cleavages between proponents and opponents of European solidarity are not noticeable [41]. This is why it is also important to investigate attitudes/beliefs of young generations towards solidarity. The analyses in this study showed that not many of the background characteristics and attitudes are associated with European cooperation to ensure high levels of employment and strengthen the economies as measures of solidarity.

Lastly, in this study, the results from Norway often deviated from, or even contradicted, the results from other countries. Unlike all the other countries, Norway is part of the EEA, but not part of the EU. However, further investigation on this is necessary.

It needs to be acknowledged that the analyses in this article are subject to some limitations. An attempt was made to look at more detailed characteristics of participating European countries in the ICCS 2016 while taking into account the topic of unemployment insurance benefits (amount and duration of benefits), supposing that this information could be associated with personal views on unemployment. The content from the Mutual Information System for Social Protection (MISSOC) database was analysed, taking the most up-to-date data from 1 January 2021 [42]. The analysis showed that national systems vary substantially. For example, fixed amount vs. varied amount of benefits or combinations of both, as well the fact that some countries set a minimum, some a maximum, and other both. The durations of unemployment benefits also vary significantly across countries: there is either a fixed flat rate or a variety of flat rates, and even combinations thereof, and in some cases, when calculating the amount, personal circumstances are also taken into account. Therefore, the analysis based on these sources could not be performed, as the systems are too complex and way too diverse and depend on too many characteristics at the individual level, which makes comparisons impossible. This does not permit taking this information into account to interpret the results of the analyses in this study. The same applies to other macroeconomic indicators, like GDP (due to very different living standards in the participating countries) and unemployment rate (due to different rates related with the age groups). This is why our secondary analyses are done from only one source, the ICCS international database, and used the available SES indicator as home predictor associated with attitudes towards future unemployment.

## Figures and Tables

**Table 1:** Description of variables and analysis plan.

Topic	Statements	Response Categories	Further Analysis Plan/Predictors
A. Students’ perception on their individual future related to employment	1. I will find a steady job; 2. My financial situation will be better than that of my parents; 3. I will find a job I like; 4. I will earn enough money to start a family.	1—Very likely 2—Likely 3—Unlikely 4—Very unlikely	The four statements will be used to construct a scale on the students’ “Financial wellbeing expectations for the future”. This scale will be used to test its relationship with the following variables: gender (male and female); immigration background (at least one parent born in the country, students born in the country but parents born abroad, and both students and parents born abroad); students’ future expected educational attainment (ISCED); students’ family SES; students’ civic knowledge scores; and students’ endorsements of EU cooperation scale.
B. Students’ attitudes towards future unemployment in Europe	1. The economy will be weaker in all European countries; and 2. There will be a rise in poverty and unemployment in Europe.	1—Very likely 2—Likely 3—Unlikely 4—Very unlikely	The two variables (statements) will be dichotomised, so that “Unlikely” and “Very unlikely” will become the first category while “Very likely” and “Likely” will become the second category. Each of the dichotomised variables will be used as dependent ones in binary logistic regression with the following predictors: gender, immigration background; expected ISCED; family SES; students’ endorsements of EU cooperation scale; and students’ civic knowledge.
C. Students’ attitudes towards European cooperation to guarantee high levels of employment and strengthen the economy	1. European countries should cooperate to guarantee high levels of employment; 2. European countries should cooperate to strengthen their economies.	1—Strongly agree 2—Agree 3—Disagree 4—Strongly disagree	The two variables (statements) will be dichotomised, so that “Disagree” and “Strongly disagree” will become the first category while “Agree” and “Strongly agree” will become the second category. Each of the dichotomised variables will be used as dependent ones in binary logistic regression with the following predictors: gender; immigration background; expected further education; family SES; and students’ civic knowledge. These analyses would not use the “Students’ endorsement on European cooperation” scale because the same items used as dependent variables were also used to produce this scale and would add collinearity to the models.

**Table 2:** Factor loadings from the PCA for the “Financial wellbeing expectations for the future” scale.

Component	Initial Eigenvalues			Extraction Sums of Squared Loadings		
	Total	Percent of Variance	Cumulative Percent	Total	Percent of Variance	Cumulative Percent
1	2.709	54.179	54.179	2.709	54.179	54.179
2	0.738	14.769	68.949			
3	0.587	11.734	80.683			
4	0.491	9.829	90.511			
5	0.474	9.489	100.000			

**Table 3:** Education systems’ averages on the “Financial wellbeing expectations for the future” scale.

Education System	Mean	(SE)
Belgium (Flemish)	49.77	(0.33)
Bulgaria	48.11	(0.30)
Croatia	48.65	(0.30)
Denmark	53.44	(0.29)
Estonia	50.46	(0.21)
Finland	50.15	(0.22)
Germany, North-Rhine Westphalia	53.30	(0.36)
Italy	51.68	(0.23)
Latvia	52.68	(0.27)
Lithuania	51.65	(0.26)
Malta	52.28	(0.16)
Netherlands	53.20	(0.26)
Norway	50.50	(0.22)
Slovenia	51.04	(0.23)
Sweden	50.44	(0.18)
ICCS European average	51.16	(0.07)

## Data Availability

Publicly available datasets were analysed in this study. These data can be found here: https://www.iea.nl/data-tools/repository/iccs (accessed on 22 December 2021).

## Supplementary Materials

The following supporting information can be downloaded at: https://www.mdpi.com/article/10.3390/ejihpe12020017/s1, Table S1. Students expectations to their individual future – job (very likely or likely); Table S2. Students expectations to their individual future – financial situation (very likely or likely); Table S3. Students’ negative expectations about the future of Europe (very likely or likely); Table S4. Students’ attitudes towards cooperation among European countries to guarantee high levels of employment and to strengthen their economies (strongly agree or agree); Table S5. Results from the binary logistic regression using “The economy will be weaker in all European countries” and selected predictors; Table S6. Results from the binary logistic regression using “There will be a rise in poverty and unemployment in Europe” and selected predictors; Table S7. Results from the binary logistic regression using “European cooperation to guarantee high levels of employment” and selected predictors; Table S8. Results from the binary logistic regression using “European countries should cooper-ate to strengthen their economies” and selected predictors.

## References

[1] WCED—United Nations World Commission on Environment and Development (1987). Our Common Future: Report of the World Commission on Environment and Development (Brundtland Report).

[2] Harris J.M., Goodwin N.R., Harris J.M., Wise T.A., Gallagher K., Goodwin N.R. (2001). Volume Introduction. A Survey of Sustainable Development: Social And Economic Dimensions.

[3] United Nations, Department of Economic and Social Affairs THE 17 GOALS—Sustainable Development. https://sdgs.un.org/goals.

[4] Seghezzo L. (2009). The Five Dimensions of Sustainability. Environ. Politics.

[5] (2019). Team Prakati Dimensions of Sustainability.

[6] United Nations Human Rights Office of the High Commissioner International Cooperation and Solidarity. https://www.ohchr.org/EN/Issues/Development/Pages/InternationalCooperationSolidarity.aspx.

[7] Losito B., Agrusti G., Damiani V., Schulz W. (2018). Young People’s Perceptions of Europe in a Time of Change.

[8] World Economic Forum (2016). Europe: What to Watch out for in 2016–2017 (Global Agenda Council on Europe).

[9] Defourny E., Maggio L., Schulmeister P. (2018). Delivering on Europe: Citizens’ Views on Current and Future EU Action.

[10] European Commission Eurobarometer 89.2 (2018): One Year to Go to the European Elections 2018. https://www.da-ra.de/dara/study/web_show?res_id=678315&lang=en&mdlang=en&detail=true.

[11] European Commission (2016). Europeans in 2016: Perceptions and Expectations, the Fight Against Terrorism….

[12] Ciornei I., Ross M.G. (2021). Solidarity in Europe: From Crisis to Policy?. Acta Polit..

[13] Archick K. (2018). The European Union: Ongoing Shallenges and Future Prospects.

[14] European Parliament An Economy That Works for People: Temporary Support to Mitigate Unemployment Risks in an Emergency (SURE) 2021. https://www.europarl.europa.eu/legislative-train/api/stages/report/current/theme/an-economy-that-works-for-people/file/coronavirus-sure-eu-support-to-mitigate-unemployment-risks-in-emergency.

[15] von der Leyen U., European Commission, Directorate General for Communication (2019). A Union That Strives for More: My Agenda for Europe.

[16] Schmid G. (2020). Beyond European Unemployment Insurance. Less Moral Hazard, More Moral Assurance?. Transf. Eur. Rev. Labour Res..

[17] Kuhn T., Nicoli F., Vandenbroucke F. (2020). Preferences for European Unemployment Insurance: A Question of Economic Ideology or EU Support?. J. Eur. Public Policy.

[18] Cebolla-Boado H., Miyar-Busto M., Muñoz-Comet J. (2019). How Much Can You Take with You? The Role of Education in Explaining Differences in the Risk of Unemployment between Migrants and Natives. CMS.

[19] Federico V., Maggini N., Federico V., Lahusen C. (2018). Disability, Unemployment, Immigration: Does Solidarity Matter at the Times of Crisis in Italy?. Solidarity as a Public Virtue? Law and Public Policies in the European Union.

[20] Kenfack C.E. (2018). Changing Environment, Just Transition and Job Creation: Perspectives from the South.

[21] Lallement M., Serrano-Pascual A., Jepsen M. (2019). Can We Still Speak the Language of Unemployment? Some Reflections Based on the French Experience. The Deconstruction of Employment as a Political Question.

[22] McFarland J., Hassar B., de Brey C., Snyder T., Wang X., Wilkinson-Flicker S., Gebrekristos S., Zhang J., Rathbun A., Barmer A. (2017). The Condition of Education 2017 (NCES 2017-144).

[23] Bonanomi A., Rosina A. (2020). Employment Status and Well-Being: A Longitudinal Study on Young Italian People. Soc. Indic. Res..

[24] Corrales-Herrero H., Rodriguez-Prado B. (2021). Measuring Youth Living Conditions in Europe: A Multidimensional Cross-Country Approach. Soc. Indic. Res..

[25] de Brey C., Snyder T.D., Zhang A., Dillow S.A. (2021). Digest of Education Statistics 2019.

[26] Ormiston R. (2016). Does High School Employment Develop Marketable Skills?. J. Labor Res..

[27] Snyder T.D., de Brey C., Dillow S.A. (2019). Digest of Education Statistics 2018.

[28] Snyder T.D., de Brey C., Dillow S.A. (2019). Digest of Education Statistics 2017.

[29] Schulz W., Fraillon J., Ainley J., Losito B., Kerr D. (2008). International Civic and Citizenship Education Study: Assessment Framework.

[30] Schulz W., Ainley J., Fraillon J., Kerr D., Losito B. (2010). ICCS 2009 International Report: Civic Knowledge, Attitudes, and Engagement among Lower-Secondary Students in 38 Countries.

[31] Schulz W., Fraillon J., Ainley J., Losito B., Kerr D. (2016). IEA International Civic and Citizenship Education Study 2016: Assessment Framework.

[32] Schulz W., Ainley J., Fraillon J., Losito B., Agrusti G., Friedman T. (2018). Becoming Citizens in a Changing World: IEA International Civic and Citizenship Education Study 2016 International Report.

[33] Schulz W., Carstens R., Losito B., Fraillon J. (2018). ICCS 2016 Technical Report.

[34] Köhler H., Weber S., Brese F., Schulz W., Carstens R. (2018). ICCS 2016 User Guide for the International Database.

[35] International Association for the Evaluation of Educational Achievement ICCS 2016 International Database. https://www.iea.nl/data-tools/repository/iccs.

[36] von Davier M., Gonzalez E., Mislevy R. (2009). What Are Plausible Values and Why Are They Useful?. IERI Monogr. Ser..

[37] Mirazchiyski P.V. (2021). RALSA: The R Analyzer for Large-scale Assessments. Large-Scale Assess. Educ..

[38] Vasilopoulou S., Talving L. (2020). Poor versus Rich Countr.ries: A Gap in Public Attitudes towards Fiscal Solidarity in the EU. West. Eur. Politics.

[39] Franchino F., Segatti P. (2019). Public Opinion on the Eurozone Fiscal Union: Evidence from Survey Experiments in Italy. J. Eur. Public Policy.

[40] GDP—Current US$ Data. https://data.worldbank.org/indicator/NY.GDP.MKTP.CD?end=2016&most_recent_value_desc=true&start=2016.

[41] Gerhards J., Lengfeld H., Ignácz S.Z., Kley K.F., Priem M., Gerhards J., Lengfeld H., Ignácz S.Z., Priem M. (2019). Theoretical Framework—Conceptualising and Understanding European Solidarity. European Solidarity in Times of Crisis: Insights from a Thirteen-Country Survey.

[42] MISSOC Database. https://www.missoc.org/missoc-database/comparative-tables/.

