# Supplementary material for: Attitudes towards Future Unemployment and European Cooperation to Reduce Unemployment among 8th Graders in EU/European Countries
Source: Eur J Investig Health Psychol Educ. 2022 Feb 17;12(2):218–35. doi: 10.3390/ejihpe12020017 (PMC8870811; doi:10.3390/ejihpe12020017)
Supplement: Supplementary file 1 [file ejihpe-12-00017-s001.zip › ejihpe-1543129-supplementary.pdf]

European Journal of
Investigation in Health, Psychology and Education

MDPI

Supplementary material – set of topics, variables and results

Systematic literature review to identify relevant predictors

The systematic literature review was performed using four different databases: JSTOR [18], ERIC [19], SpringerLink [20] and SAGE Open [21]. Several relevant descriptors were used (see below) The sources focused on university students are out of scope and were excluded them from searching terms. Only sources in English language were considered and both quantitative and qualitative studies' literature sources were reviewed. There were no specific restrictions on the studies involved, so book sections were used as well as articles published in professional and academic journals. The final search and review of the studies was carried out in August 2021. The time period was limited between 2016 (year of data collection for ICCS 2016 cycle) and 2021, except in the ERIC database (2017-2021), where precise time-limiting option does not exist.

In the first phase of searching the following descriptors were used and the search for the literature applied three formulas:

1. (attitudes OR beliefs) AND unemployment AND students -university students
2. (attitudes OR belief) AND (financial support OR financial aid) AND students -university students
3. risk groups AND unemployment AND students -university students

The results identified in the first phase were 4,305 freely accessible items (the number of all items, regardless of the access option, was 16,873). More specifically:

- JSTOR:
  a. (attitudes OR beliefs) AND unemployment AND students -university students: 102 (844)
  b. (attitudes OR belief) AND (financial support OR financial aid) AND students -university students: 137 (1331)
  c. risk groups AND unemployment AND students -university students: 157 (782)
- ERIC:
  a. (attitudes OR beliefs) AND unemployment AND students -university students: 26 (48)
  b. (attitudes OR belief) AND (financial support OR financial aid) AND students -university students: 2000 (7402)
  c. risk groups AND unemployment AND students -university students: 44 (69)
- SpringerLink:
  a. (attitudes OR beliefs) AND unemployment AND students -university students: 334 (579)
  b. (attitudes OR belief) AND (financial support OR financial aid) AND students -university students: 397 (741)
  c. risk groups AND unemployment AND students -university students: 429 (708)
- SAGE Open:
  a. (attitudes OR beliefs) AND unemployment AND students -university students: 128 (1089)
  b. (attitudes OR belief) AND (financial support OR financial aid) AND students -university students: 409 (2302)
  c. risk groups AND unemployment AND students -university students: 142(978)

The four different databases don't have the same search options, so the search specifications (conditions) were adapted to the individual database. Items without full access (12,568 in total) were excluded. In JSTOR the following subjects were chosen to narrow the search: African American studies, American studies, Asian studies, anthropology, business, economics, education, labor and employment relations, philosophy, political

science and population studies, and sociology. When the first search formula was used, 35 results were identified, with second formula – 54 results, and with third – 44 results. The search in ERIC was narrowed to the following descriptors: secondary education, elementary education, high schools, middle schools, junior high schools, intermediate grades, and primary education. In this database just six articles were found with the aforementioned search terms. In SpringerLink it is also possible to choose different disciplines to search the literature and the search was limited to the following: social science, economics, psychology and education. With first formula 154 items were found, with the second – 130, and with third – 192. The search was performed for each discipline separately because there was no option to use several disciplines at the same time. In Sage Open the following disciplines were selected for the search: communication and media studies, criminology and criminal justice, education, psychology and counselling, and sociology. When the first formula was applied 13 results were identified, with second formula – 16, and with last one – 9 items.

In this phase a total of 653 items of literature were found using the search terms from above. In the next step the abstracts of the sources were read and 28 relevant items were selected. Next, five duplicates were excluded, and then from the remaining 23 items 12 excluded because they did not satisfy our criteria.

Statistical analyses

Topic S1: Students' perception on their individual future related to employment and income

The ICCS 2016 European regional questionnaire contains two questions asking students about their expectations in relation to their future job opportunities: (1) I will find a steady job; and (2) I will find a job I like. In addition, the database contains data from the following two statements related with the student opinion on their future financial wellbeing: (1) My financial situation will be better than that of my parents; and (2) I will earn enough money to start a family. The four statements have the following response options: (1) – Very likely; (2) Likely; (3) Unlikely; (4) Very unlikely. Tables A1 and A2 contain the percentages of students who tend to agree with these four statements, i.e. choosing the options "Very likely" or "Likely".

Topic S2: Students' perception on the future European economy, poverty and unemployment Europe

Another set of analyses is devoted to students' perceptions of Europe's future with focus on negative scenarios: (1) The economy will be weaker in all European countries; and (2) There will be rise of poverty and unemployment in Europe. The two statements have the following response options: (1) Very likely; (2) Likely; (3) Unlikely; (4) Very unlikely. Table B contains the percentages of students who tend to agree with these statements, i.e. choosing the options "Very likely" or "Likely".

Topic S3: Student's attitudes towards European cooperation to guarantee high levels of employment and strengthen the economies

The ICCS 2016 European regional questionnaire included a set of eight items investigating students' attitudes toward cooperation among European countries. This question sought to capture students' views on the adoption of common policies in Europe (e.g., environmental policies) or on cooperation in specific areas, like strategies to reduce unemployment and to address economic crises. The analyses in this article use two of the statements: (1) European countries should cooperate to guarantee high levels of employment; and (2) European countries should cooperate to strengthen their economies. The response categories are (1) Strongly agree; (2) Agree; (3) Disagree; (4) Strongly disagree. Table C reports percentages of students choosing the first two categories ("Strongly agree" and "Agree").

Topic S4: Predictors of student anticipation that the economy will be weaker in all European countries

The statement that "The economy will be weaker in all European countries" from the ICCS 2016 European Module has four categories: (1) Very likely; (2) Likely; (3) Unlikely; (4) Very unlikely. These categories were collapsed into two: (1) Very unlikely or unlikely; and (2) Very likely or likely. The data from the recoded answers on this statement were used as dependent variable in a binary logistic regression model. The purpose of this model is to identify the strongest predictors of the student opinion that the economy in all European countries will be weaker. The results are presented in Table D.

Topic S5: Predictors of student anticipation that there be rise of poverty and unemployment in Europe

The statement that "There will be a rise in poverty and unemployment in Europe" from the ICCS 2016 European Module has four categories: (1) Very likely; (2) Likely; (3) Unlikely; (4) Very unlikely. These categories were collapsed into two: (1) Very unlikely or unlikely; and (2) Very likely or likely. The data from the recoded answers on this statement were used as dependent variable in a binary logistic regression model. The purpose of this model is to identify the strongest predictors of the student opinion that there will be rise of poverty and unemployment in Europe. The results are presented in Table E.

Topic S6: Predictors of student agreement that European cooperation is needed to guarantee high levels of employment

The statement that "European cooperation to guarantee high levels of employment" from the ICCS 2016 European Module has four categories: (1) Strongly agree; (2) Agree; (3) Disagree; (4) Strongly disagree. These categories were collapsed into two: (1) Strongly disagree or disagree; and (2) Strongly agree or agree. The data from the recoded answers on this statement were used as dependent variable in a binary logistic regression model. The purpose of this model is to identify the strongest predictors of the student agreement that there should be European cooperation to guarantee high levels of employment. The results are presented in Table F.

Topic S7: Predictors of student agreement that European cooperation is needed strengthen the economies

The statement that "European countries should cooperate to strengthen their economies" from the ICCS 2016 European Module has four categories: (1) Strongly agree; (2) Agree; (3) Disagree; (4) Strongly disagree. These categories were collapsed into two: (1) Strongly disagree or disagree; and (2) Strongly agree or agree. The data from the recoded answers on this statement were used as dependent variable in a binary logistic regression model. The purpose of this model is to identify the strongest predictors of the student agreement that there should be European cooperation to strengthen the economies. The results are presented in Table G.

Tables' legends

Some of the tables contain certain characters to designate specific properties of the results. Please refer to the list below for explanation on the meaning of these symbols.

( )	Standard errors appear in parentheses
1	Significantly higher than European ICCS average
2	Significantly lower than European ICCS average
3	Significantly higher by 10 percentage points than the European ICCS average
4	Significantly lower by 10 percentage points than the European ICCS average
*	Benchmarking participants not reaching the sample participation requirements
**	National defined population covers 90% to 95% of national target population
***	Met guidelines for sampling participation rates only after replacement schools were included
+	Country sampled grade 9 students

European Journal of Investigation in Health, Psychology and Education

MDPI

Table S1.1. Students expectations to their individual future – job (very likely or likely).

Country	I will find a steady job						I will find a job I like					
	Sampled cases	Population estimate		Percentages		Difference with ICCS European average	Sam-pled cases	Population estimate		Percentages		Difference with ICCS European average
Belgium (Flemish)	2859	70712.36	(2365.69)	97.98	(0.32)	1	2742	67973.64	(2286.54)	94.39	(0.46)	1
Bulgaria	2681	47595.15	(1161.44)	91.51	(0.62)	2	2487	44111.53	(1124.24)	85.62	(0.86)	2
Croatia	3462	33971.49	(745.03)	89.74	(0.67)	2	3248	31946.81	(734.58)	84.47	(0.93)	2
Denmark ***	5705	48891.01	(1250.60)	98.25	(0.16)	1	5691	48854.23	(1237.87)	98.18	(0.24)	1
Estonia **	2694	9826.35	(234.46)	94.74	(0.49)		2622	9616.16	(249.91)	92.76	(0.50)	1
Finland	3054	50350.79	(870.47)	97.21	(0.32)	1	2927	48320.47	(854.22)	93.57	(0.45)	1
Germany, North-Rhine Westphalia	1396	143971.97	(4650.10)	96.90	(0.61)		1331	137916.27	(4805.77)	93.66	(0.97)	
Italy	3160	509436.24	(9871.59)	92.23	(0.53)	2	3036	489856.33	(9719.12)	88.70	(0.54)	2
Latvia ***	3042	15616.24	(296.36)	95.89	(0.52)		2865	14715.26	(300.44)	90.46	(0.60)	
Lithuania	3491	24768.54	(396.80)	97.04	(0.36)	1	3265	23063.65	(387.63)	90.83	(0.58)	
Malta	3429	3598.80	(34.34)	92.70	(0.44)	2	3291	3453.35	(39.08)	89.35	(0.54)	2
Netherlands ***	2710	166652.39	(5558.45)	97.33	(0.34)	1	2669	164071.82	(5595.90)	95.98	(0.53)	1
Norway + **	6015	53753.65	(1178.22)	98.00	(0.21)	1	5876	52575.24	(1176.05)	96.52	(0.27)	1
Slovenia	2605	15885.06	(365.56)	92.02	(0.55)	2	2487	15187.09	(355.02)	88.30	(0.70)	2
Sweden **	3031	96486.47	(4647.39)	95.69	(0.38)		2829	89483.27	(3612.97)	89.45	(1.01)	2
ICCS European average	-	-	-	95.15	(0.12)	-	-	-	-	91.48	(0.17)	-

Table S1.2. Students expectations to their individual future – financial situation (very likely or likely).

Country	My financial situation will be better than that of my parents				I will earn enough money to start a family			
	Sampled cases	Population estimate	Percentages	Difference with ICCS European average	Sampled cases	Population estimate	Percentages	Difference with ICCS European average
Belgium (Flemish)	1907	48136.57 (1998.83)	68.33 (1.08)	4	2850	70750.49 (2466.12)	98.26 (0.28)	1
Bulgaria	2467	43672.61 (1124.45)	84.33 (0.81)	1	2644	46897.45 (1267.28)	90.41 (0.93)	2
Croatia	2922	28522.58 (657.53)	75.62 (0.99)	2	3629	35713.92 (651.52)	94.39 (0.37)	2
Denmark ***	4722	40680.60 (1093.59)	84.03 (0.64)	1	5698	48909.83 (1233.30)	98.38 (0.22)	1
Estonia **	2384	8702.69 (231.80)	84.35 (0.79)	1	2706	9884.45 (243.07)	95.36 (0.37)	
Finland	2368	38934.33 (783.89)	76.34 (0.86)	2	3013	49682.50 (867.80)	96.13 (0.36)	
Germany, North-Rhine Westphalia	1031	106511.85 (3878.63)	73.83 (1.66)		1383	142030.82 (4564.71)	95.90 (0.72)	
Italy	2775	447486.27 (9652.93)	81.28 (0.76)	1	3240	521980.13 (10382.60)	94.60 (0.56)	
Latvia ***	2743	14035.39 (266.74)	86.55 (0.76)	1	3012	15497.89 (295.11)	95.38 (0.41)	
Lithuania	3086	21861.34 (391.82)	86.23 (0.69)	1	3494	24726.27 (392.46)	96.90 (0.40)	1
Malta	3121	3276.75 (41.42)	84.79 (0.62)	1	3416	3584.68 (37.28)	92.22 (0.50)	2
Netherlands ***	1955	120822.16 (4673.73)	71.22 (1.16)	2	2732	167629.55 (5539.17)	97.93 (0.41)	1
Norway + **	4456	40032.07 (978.43)	74.70 (0.65)	2	5907	52864.67 (1159.95)	96.81 (0.31)	1
Slovenia	1994	12232.65 (337.76)	71.19 (1.06)	2	2694	16452.88 (380.49)	95.39 (0.47)	
Sweden **	2164	67757.20 (2868.17)	67.70 (1.18)	4	3062	97494.99 (4678.54)	96.06 (0.42)	
ICCS European average	-	- -	78.03 (0.25)	-	-	- -	95.61 (0.12)	-

Table S2. Students' negative expectations about the future of Europe (very likely or likely).

Country	The economy will be weaker in all European Countries				There will be a rise in poverty and unemployment in Europe			
	Sampled cases	Population estimate	Percentages	Difference with ICCS European average	Sampled cases	Population estimate	Percentages	Difference with ICCS European average
Belgium (Flemish)	1215	30854.31 (1443.16)	43.24 (1.30)		1602	39582.81 (1496.46)	55.21 (1.23)	1
Bulgaria	1309	23680.50 (768.19)	46.05 (1.08)	1	1717	30310.94 (871.96)	59.01 (1.23)	1
Croatia	1510	14516.82 (412.19)	38.54 (0.97)	2	2078	20064.21 (564.15)	53.20 (1.13)	
Denmark ***	1703	14748.57 (557.01)	30.02 (0.85)	4	2084	17745.88 (569.06)	36.12 (0.87)	4
Estonia **	1183	4344.19 (140.89)	42.24 (0.94)		1387	5047.59 (147.28)	49.04 (0.85)	2
Finland	1407	23172.48 (598.48)	45.05 (0.92)	1	1475	24287.21 (587.07)	47.22 (0.90)	2
Germany, North-Rhine Westphalia	595	63156.37 (3487.28)	42.94 (2.04)		754	77398.25 (2946.23)	52.45 (1.60)	
Italy	1759	282573.62 (6667.44)	51.65 (1.07)	1	2167	350262.44 (7789.66)	63.70 (0.88)	3
Latvia ***	1349	7153.42 (224.38)	44.41 (1.11)		1620	8387.99 (223.37)	51.77 (0.96)	
Lithuania	1437	9648.01 (361.68)	38.05 (1.26)	2	1641	11111.59 (320.04)	43.76 (0.95)	2
Malta	1791	1891.13 (36.63)	49.50 (0.93)	1	2145	2253.68 (35.39)	58.77 (0.81)	1
Netherlands ***	975	62438.32 (3174.75)	36.52 (1.52)	2	1170	73339.04 (3193.79)	42.96 (1.21)	2
Norway + **	2957	26325.96 (640.77)	48.54 (0.65)	1	3440	30887.26 (738.89)	57.04 (0.69)	1
Slovenia	1424	8658.76 (241.69)	50.31 (1.05)	1	1874	11467.86 (290.47)	66.64 (1.04)	3
Sweden **	1123	36346.05 (2282.23)	36.68 (1.04)	2	1526	48939.95 (2798.28)	48.70 (1.16)	2
ICCS European average	-	- -	42.92 (0.30)	-	-	- -	52.37 (0.27)	-

Table S3. Students' attitudes towards cooperation among European countries to guarantee high levels of employment and to strengthen their economies (strongly agree or agree).

Country	European countries should cooperate to guarantee high levels of employment				European countries should cooperate to strengthen their economies			
	Sampled cases	Population estimate	Percentages	Difference with ICCS European average	Sampled cases	Population estimate	Percentages	Difference with ICCS European average
Belgium (Flemish)	2793	69475.41 (2448.47)	96.26 (0.42)	1	2764	68670.96 (2457.38)	95.14 (0.45)	
Bulgaria	2724	48012.09 (1211.52)	92.17 (0.66)	2	2685	47222.15 (1100.83)	91.07 (0.70)	2
Croatia	3821	37536.97 (669.00)	99.10 (0.20)	1	3765	36945.99 (641.49)	97.87 (0.29)	1
Denmark ***	5505	47085.81 (1209.55)	95.09 (0.44)		5387	46175.57 (1213.88)	92.89 (0.44)	2
Estonia **	2693	9873.02 (238.56)	95.32 (0.54)		2717	9969.63 (240.94)	96.26 (0.38)	1
Finland	3027	49931.83 (851.25)	96.78 (0.29)	1	3013	49710.83 (855.47)	96.57 (0.33)	1
Germany, North-Rhine Westphalia	1353	139758.61 (4619.20)	93.86 (0.75)		1324	136548.16 (4443.88)	91.98 (0.91)	
Italy	3151	507084.35 (9010.78)	92.21 (0.53)	2	3264	526310.62 (10334.11)	95.50 (0.43)	1
Latvia ***	2957	15221.99 (304.73)	93.58 (0.56)	2	2969	15259.85 (282.58)	93.90 (0.50)	
Lithuania	3413	24288.45 (403.25)	95.27 (0.47)		3437	24471.32 (417.73)	95.97 (0.45)	1
Malta	3543	3717.69 (36.74)	95.31 (0.41)		3445	3612.41 (38.97)	92.88 (0.51)	2
Netherlands ***	2583	158981.80 (5511.39)	92.60 (0.61)	2	2585	158951.44 (5624.92)	92.79 (0.62)	2
Norway + **	5796	51955.61 (1202.34)	94.83 (0.25)		5662	50710.59 (1135.56)	92.44 (0.39)	2
Slovenia	2738	16732.02 (366.32)	96.76 (0.43)	1	2685	16420.09 (373.88)	95.00 (0.49)	
Sweden **	2947	93503.38 (4509.77)	93.46 (0.48)	2	2975	94203.51 (4271.12)	94.27 (0.54)	
ICCS European average	-	- -	94.84 (0.13)	-	-	- -	94.30 (0.13)	-

European Journal of
Investigation in Health, Psychology and Education

MDPI

Table S4. Results from the binary logistic regression using "The economy will be weaker in all European countries" and selected predictors.

Educational system	Variable	Coefficients	(SE)	Wald Statistic	p	Odds Ratio	(SE)
Belgium (Flemish)	(Intercept)	2.59	(0.50)	5.23	0.000	13.43	(6.86)
	Endorsement of EU cooperation	0.00	(0.01)	0.76	0.447	1.00	(0.01)
	Civic knowledge	0.00	(0.00)	-6.71	0.000	1.00	(0.00)
	Gender (girls vs. boys)	0.20	(0.10)	2.03	0.042	1.22	(0.12)
	Immigration status	-0.11	(0.07)	-1.47	0.141	0.90	(0.07)
	Expected further education	-0.07	(0.06)	-1.32	0.188	0.93	(0.05)
	SES	-0.07	(0.05)	-1.28	0.199	0.94	(0.05)
Bulgaria	(Intercept)	0.25	(1.03)	0.24	0.809	1.28	(1.33)
	Endorsement of EU cooperation	0.01	(0.00)	2.88	0.004	1.01	(0.00)
	Civic knowledge	0.00	(0.00)	-6.95	0.000	1.00	(0.00)
	Gender (girls vs. boys)	0.11	(0.09)	1.18	0.238	1.12	(0.11)
	Immigration status	0.33	(0.34)	0.97	0.333	1.39	(0.48)
	Expected further education	0.00	(0.05)	-0.08	0.934	1.00	(0.05)
	SES	-0.03	(0.07)	-0.38	0.701	0.97	(0.07)
Croatia	(Intercept)	1.37	(0.52)	2.63	0.008	3.97	(2.07)
	Endorsement of EU cooperation	0.00	(0.00)	0.17	0.861	1.00	(0.00)
	Civic knowledge	0.00	(0.00)	-5.64	0.000	1.00	(0.00)
	Gender (girls vs. boys)	0.12	(0.09)	1.34	0.181	1.13	(0.10)
	Immigration status	0.22	(0.14)	1.57	0.116	1.24	(0.17)
	Expected further education	-0.03	(0.07)	-0.49	0.625	0.97	(0.06)
	SES	-0.11	(0.05)	-2.29	0.022	0.89	(0.04)
Denmark	(Intercept)	1.23	(0.51)	2.42	0.016	3.41	(1.73)
	Endorsement of EU cooperation	0.00	(0.00)	-0.17	0.869	1.00	(0.00)
	Civic knowledge	0.00	(0.00)	-5.92	0.000	1.00	(0.00)
	Gender (girls vs. boys)	0.16	(0.08)	1.84	0.066	1.17	(0.10)
	Immigration status	0.06	(0.11)	0.54	0.592	1.06	(0.12)
	Expected further education	-0.07	(0.04)	-1.74	0.081	0.93	(0.04)
	SES	-0.05	(0.05)	-1.10	0.272	0.95	(0.05)
Estonia	(Intercept)	0.62	(0.50)	1.24	0.213	1.86	(0.93)
	Endorsement of EU cooperation	0.01	(0.01)	1.76	0.079	1.01	(0.01)
	Civic knowledge	0.00	(0.00)	-2.05	0.040	1.00	(0.00)
	Gender (girls vs. boys)	0.15	(0.08)	1.87	0.062	1.17	(0.10)
	Immigration status	-0.15	(0.13)	-1.14	0.256	0.86	(0.11)
	Expected further education	-0.11	(0.05)	-2.04	0.042	0.89	(0.05)
	SES	0.07	(0.04)	1.76	0.078	1.07	(0.04)
Finland	(Intercept)	0.87	(0.55)	1.57	0.116	2.38	(1.34)
	Endorsement of EU cooperation	0.00	(0.00)	-0.80	0.422	1.00	(0.00)
	Civic knowledge	0.00	(0.00)	-2.05	0.040	1.00	(0.00)
	Gender (girls vs. boys)	0.15	(0.08)	1.89	0.059	1.16	(0.09)

Educational system	Variable	Coefficients	(SE)	Wald Statistic	p	Odds Ratio	(SE)
	Immigration status	-0.08	(0.15)	-0.52	0.606	0.92	(0.14)
	Expected further education	-0.01	(0.04)	-0.25	0.804	0.99	(0.04)
	SES	0.04	(0.04)	0.79	0.427	1.04	(0.05)
Germany, North-Rhine Westphalia	(Intercept)	1.07	(0.89)	1.21	0.226	2.94	(3.09)
	Endorsement of EU cooperation	0.01	(0.01)	1.46	0.145	1.01	(0.01)
	Civic knowledge	-0.01	(0.00)	-4.82	0.000	0.99	(0.00)
	Gender (girls vs. boys)	0.18	(0.15)	1.26	0.206	1.20	(0.18)
	Immigration status	0.14	(0.11)	1.32	0.188	1.15	(0.12)
	Expected further education	0.04	(0.07)	0.62	0.538	1.04	(0.07)
	SES	-0.08	(0.06)	-1.21	0.225	0.93	(0.06)
Italy	(Intercept)	0.37	(0.39)	0.95	0.343	1.45	(0.56)
	Endorsement of EU cooperation	0.01	(0.00)	2.87	0.004	1.01	(0.00)
	Civic knowledge	0.00	(0.00)	-3.88	0.000	1.00	(0.00)
	Gender (girls vs. boys)	0.43	(0.08)	5.62	0.000	1.53	(0.12)
	Immigration status	0.12	(0.10)	1.18	0.239	1.13	(0.11)
	Expected further education	-0.09	(0.05)	-1.68	0.092	0.92	(0.05)
	SES	-0.11	(0.04)	-2.54	0.011	0.90	(0.04)
Latvia	(Intercept)	2.37	(0.44)	5.39	0.000	10.75	(4.72)
	Endorsement of EU cooperation	0.00	(0.01)	-0.07	0.948	1.00	(0.01)
	Civic knowledge	0.00	(0.00)	-4.83	0.000	1.00	(0.00)
	Gender (girls vs. boys)	0.03	(0.10)	0.34	0.737	1.03	(0.10)
	Immigration status	-0.36	(0.15)	-2.33	0.020	0.70	(0.11)
	Expected further education	0.09	(0.07)	1.28	0.200	1.09	(0.08)
	SES	-0.03	(0.06)	-0.57	0.568	0.97	(0.06)
Lithuania	(Intercept)	1.47	(0.74)	1.99	0.047	4.34	(3.21)
	Endorsement of EU cooperation	0.00	(0.00)	0.36	0.719	1.00	(0.00)
	Civic knowledge	0.00	(0.00)	-6.47	0.000	1.00	(0.00)
	Gender (girls vs. boys)	0.12	(0.11)	1.07	0.284	1.13	(0.12)
	Immigration status	0.17	(0.22)	0.78	0.437	1.19	(0.26)
	Expected further education	-0.06	(0.07)	-0.87	0.384	0.94	(0.07)
	SES	0.02	(0.05)	0.47	0.641	1.02	(0.05)
Malta	(Intercept)	2.32	(0.34)	6.94	0.000	10.22	(3.37)
	Endorsement of EU cooperation	0.01	(0.00)	1.38	0.166	1.01	(0.00)
	Civic knowledge	-0.01	(0.00)	-10.33	0.000	0.99	(0.00)
	Gender (girls vs. boys)	0.32	(0.07)	4.44	0.000	1.38	(0.10)
	Immigration status	-0.04	(0.08)	-0.54	0.589	0.96	(0.07)
	Expected further education	-0.02	(0.04)	-0.55	0.584	0.98	(0.04)
	SES	-0.20	(0.04)	-5.55	0.000	0.82	(0.03)
Nether-lands	(Intercept)	2.04	(0.62)	3.27	0.001	7.68	(4.60)
	Endorsement of EU cooperation	0.00	(0.00)	0.37	0.713	1.00	(0.00)
	Civic knowledge	0.00	(0.00)	-5.30	0.000	1.00	(0.00)
	Gender (girls vs. boys)	0.10	(0.09)	1.03	0.303	1.10	(0.10)

Educational system	Variable	Coefficients	(SE)	Wald Statistic	p	Odds Ratio	(SE)
	Immigration status	-0.15	(0.14)	-1.02	0.308	0.86	(0.13)
	Expected further education	-0.05	(0.06)	-0.88	0.381	0.95	(0.06)
	SES	-0.08	(0.05)	-1.59	0.112	0.92	(0.05)
Norway	(Intercept)	1.12	(0.32)	3.50	0.000	3.08	(0.98)
	Endorsement of EU cooperation	0.01	(0.00)	3.48	0.000	1.01	(0.00)
	Civic knowledge	0.00	(0.00)	-6.34	0.000	1.00	(0.00)
	Gender (girls vs. boys)	0.23	(0.06)	3.55	0.000	1.25	(0.08)
	Immigration status	-0.10	(0.06)	-1.53	0.126	0.91	(0.06)
	Expected further education	0.01	(0.04)	0.30	0.763	1.01	(0.04)
	SES	0.03	(0.03)	0.89	0.372	1.03	(0.03)
Slovenia	(Intercept)	0.90	(0.43)	2.07	0.039	2.47	(1.07)
	Endorsement of EU cooperation	0.01	(0.00)	2.27	0.023	1.01	(0.00)
	Civic knowledge	0.00	(0.00)	-3.71	0.000	1.00	(0.00)
	Gender (girls vs. boys)	0.36	(0.09)	4.02	0.000	1.44	(0.13)
	Immigration status	0.09	(0.08)	1.09	0.275	1.10	(0.09)
	Expected further education	-0.08	(0.07)	-1.13	0.260	0.93	(0.06)
	SES	-0.08	(0.05)	-1.71	0.086	0.92	(0.04)
Sweden	(Intercept)	0.69	(0.40)	1.73	0.083	1.99	(0.81)
	Endorsement of EU cooperation	0.01	(0.01)	1.67	0.096	1.01	(0.01)
	Civic knowledge	0.00	(0.00)	-5.36	0.000	1.00	(0.00)
	Gender (girls vs. boys)	0.15	(0.10)	1.57	0.116	1.16	(0.11)
	Immigration status	0.11	(0.08)	1.47	0.142	1.12	(0.08)
	Expected further education	-0.06	(0.06)	-1.09	0.274	0.94	(0.05)
	SES	-0.09	(0.05)	-1.88	0.060	0.92	(0.04)

Table S5. Results from the binary logistic regression using "There will be a rise in poverty and unemployment in Europe" and selected predictors.

Educational system	Variable	Coefficients	(SE)	Wald Statistic	p	Odds Ratio	(SE)
Belgium (Flemish)	(Intercept)	-0.28	(0.54)	-0.52	0.600	0.76	(0.40)
	Endorsement of EU cooperation	0.00	(0.00)	0.49	0.624	1.00	(0.00)
	Civic knowledge	0.00	(0.00)	-0.27	0.786	1.00	(0.00)
	Gender (girls vs. boys)	0.14	(0.08)	1.73	0.084	1.15	(0.09)
	Immigration status	0.19	(0.10)	1.95	0.051	1.21	(0.12)
	Expected further education	-0.04	(0.06)	-0.71	0.479	0.96	(0.05)
	SES	0.04	(0.06)	0.69	0.492	1.04	(0.06)
Bulgaria	(Intercept)	-0.48	(1.07)	-0.45	0.652	0.62	(0.67)
	Endorsement of EU cooperation	0.01	(0.00)	1.67	0.094	1.01	(0.00)
	Civic knowledge	0.00	(0.00)	-1.32	0.185	1.00	(0.00)
	Gender (girls vs. boys)	0.23	(0.09)	2.46	0.014	1.26	(0.12)
	Immigration status	0.25	(0.35)	0.72	0.471	1.29	(0.45)
	Expected further education	0.00	(0.05)	0.09	0.932	1.00	(0.05)
	SES	-0.12	(0.06)	-2.12	0.034	0.89	(0.05)
Croatia	(Intercept)	0.13	(0.54)	0.23	0.815	1.15	(0.62)
	Endorsement of EU cooperation	-0.01	(0.01)	-1.16	0.246	0.99	(0.01)
	Civic knowledge	0.00	(0.00)	-2.05	0.041	1.00	(0.00)
	Gender (girls vs. boys)	0.43	(0.09)	4.94	0.000	1.53	(0.13)
	Immigration status	0.34	(0.11)	3.19	0.001	1.41	(0.15)
	Expected further education	0.04	(0.07)	0.60	0.547	1.04	(0.07)
	SES	-0.10	(0.05)	-1.93	0.053	0.91	(0.05)
Denmark	(Intercept)	0.84	(0.39)	2.14	0.033	2.32	(0.92)
	Endorsement of EU cooperation	-0.01	(0.00)	-1.51	0.131	0.99	(0.00)
	Civic knowledge	0.00	(0.00)	-3.73	0.000	1.00	(0.00)
	Gender (girls vs. boys)	0.11	(0.07)	1.59	0.113	1.12	(0.08)
	Immigration status	0.08	(0.09)	0.89	0.375	1.09	(0.10)
	Expected further education	-0.10	(0.05)	-2.13	0.033	0.91	(0.04)
	SES	-0.05	(0.03)	-1.37	0.171	0.95	(0.03)
Estonia	(Intercept)	0.78	(0.48)	1.61	0.107	2.18	(1.07)
	Endorsement of EU cooperation	0.00	(0.00)	0.30	0.761	1.00	(0.00)
	Civic knowledge	0.00	(0.00)	-1.31	0.191	1.00	(0.00)
	Gender (girls vs. boys)	0.41	(0.09)	4.47	0.000	1.50	(0.14)
	Immigration status	-0.14	(0.12)	-1.16	0.245	0.87	(0.10)
	Expected further education	-0.07	(0.04)	-1.67	0.094	0.93	(0.04)
	SES	0.00	(0.04)	-0.06	0.951	1.00	(0.04)
Finland	(Intercept)	0.18	(0.58)	0.31	0.755	1.20	(0.72)
	Endorsement of EU cooperation	0.00	(0.00)	0.17	0.862	1.00	(0.00)
	Civic knowledge	0.00	(0.00)	-1.96	0.050	1.00	(0.00)
	Gender (girls vs. boys)	0.26	(0.10)	2.59	0.010	1.30	(0.13)
	Immigration status	0.06	(0.14)	0.44	0.659	1.06	(0.15)

Educational system	Variable	Coefficients	(SE)	Wald Statistic	p	Odds Ratio	(SE)
	Expected further education	0.01	(0.04)	0.17	0.864	1.01	(0.04)
	SES	-0.07	(0.04)	-1.71	0.087	0.93	(0.04)
Germany, North-Rhine Westphalia	(Intercept)	0.29	(0.53)	0.56	0.575	1.34	(0.72)
	Endorsement of EU cooperation	0.01	(0.01)	1.98	0.048	1.01	(0.01)
	Civic knowledge	0.00	(0.00)	-2.56	0.010	1.00	(0.00)
	Gender (girls vs. boys)	-0.06	(0.13)	-0.46	0.649	0.94	(0.12)
	Immigration status	0.09	(0.11)	0.75	0.452	1.09	(0.13)
	Expected further education	0.01	(0.07)	0.15	0.882	1.01	(0.07)
	SES	-0.05	(0.06)	-0.71	0.478	0.96	(0.06)
Italy	(Intercept)	1.05	(0.38)	2.74	0.006	2.85	(1.11)
	Endorsement of EU cooperation	0.01	(0.00)	2.48	0.013	1.01	(0.00)
	Civic knowledge	0.00	(0.00)	-3.53	0.000	1.00	(0.00)
	Gender (girls vs. boys)	0.40	(0.08)	5.05	0.000	1.49	(0.12)
	Immigration status	-0.01	(0.08)	-0.07	0.946	0.99	(0.08)
	Expected further education	-0.04	(0.05)	-0.78	0.433	0.96	(0.04)
	SES	-0.17	(0.04)	-4.20	0.000	0.85	(0.03)
Latvia	(Intercept)	2.41	(0.60)	3.99	0.000	11.14	(6.43)
	Endorsement of EU cooperation	0.00	(0.01)	-0.57	0.568	1.00	(0.01)
	Civic knowledge	0.00	(0.00)	-3.23	0.001	1.00	(0.00)
	Gender (girls vs. boys)	0.23	(0.09)	2.66	0.008	1.26	(0.11)
	Immigration status	-0.49	(0.18)	-2.71	0.007	0.61	(0.11)
	Expected further education	0.06	(0.07)	0.79	0.431	1.06	(0.08)
	SES	-0.02	(0.06)	-0.28	0.778	0.98	(0.06)
Lithuania	(Intercept)	1.59	(0.66)	2.42	0.015	4.92	(3.44)
	Endorsement of EU cooperation	-0.01	(0.00)	-2.34	0.019	0.99	(0.00)
	Civic knowledge	0.00	(0.00)	-4.95	0.000	1.00	(0.00)
	Gender (girls vs. boys)	0.45	(0.07)	6.44	0.000	1.57	(0.11)
	Immigration status	0.10	(0.18)	0.54	0.586	1.10	(0.20)
	Expected further education	-0.06	(0.06)	-0.98	0.328	0.94	(0.06)
	SES	0.01	(0.05)	0.18	0.859	1.01	(0.05)
Malta	(Intercept)	1.67	(0.30)	5.63	0.000	5.34	(1.61)
	Endorsement of EU cooperation	0.01	(0.00)	2.04	0.042	1.01	(0.00)
	Civic knowledge	0.00	(0.00)	-5.60	0.000	1.00	(0.00)
	Gender (girls vs. boys)	0.25	(0.07)	3.52	0.000	1.28	(0.09)
	Immigration status	-0.11	(0.07)	-1.48	0.140	0.90	(0.07)
	Expected further education	-0.05	(0.04)	-1.12	0.262	0.96	(0.04)
	SES	-0.17	(0.04)	-4.39	0.000	0.84	(0.03)
Netherlands	(Intercept)	0.98	(0.48)	2.04	0.042	2.66	(1.25)
	Endorsement of EU cooperation	0.00	(0.00)	-0.54	0.588	1.00	(0.00)
	Civic knowledge	0.00	(0.00)	-2.48	0.013	1.00	(0.00)
	Gender (girls vs. boys)	-0.01	(0.10)	-0.11	0.910	0.99	(0.10)
	Immigration status	0.00	(0.10)	-0.03	0.978	1.00	(0.10)

Educational system	Variable	Coefficients	(SE)	Wald Statistic	p	Odds Ratio	(SE)
	Expected further education	-0.05	(0.06)	-0.87	0.386	0.95	(0.06)
	SES	-0.10	(0.05)	-2.01	0.045	0.91	(0.04)
Norway	(Intercept)	1.11	(0.32)	3.43	0.001	3.04	(0.99)
	Endorsement of EU cooperation	0.00	(0.00)	1.33	0.183	1.00	(0.00)
	Civic knowledge	0.00	(0.00)	-3.43	0.001	1.00	(0.00)
	Gender (girls vs. boys)	0.18	(0.06)	2.98	0.003	1.20	(0.07)
	Immigration status	-0.05	(0.06)	-0.78	0.436	0.95	(0.06)
	Expected further education	-0.02	(0.05)	-0.41	0.679	0.98	(0.05)
	SES	0.00	(0.04)	0.10	0.924	1.00	(0.04)
Slovenia	(Intercept)	-0.46	(0.44)	-1.06	0.288	0.63	(0.28)
	Endorsement of EU cooperation	0.00	(0.00)	-0.78	0.434	1.00	(0.00)
	Civic knowledge	0.00	(0.00)	0.65	0.519	1.00	(0.00)
	Gender (girls vs. boys)	0.52	(0.08)	6.24	0.000	1.69	(0.14)
	Immigration status	0.35	(0.11)	3.30	0.001	1.42	(0.15)
	Expected further education	-0.04	(0.06)	-0.71	0.481	0.96	(0.06)
	SES	-0.14	(0.04)	-3.18	0.001	0.87	(0.04)
Sweden	(Intercept)	0.65	(0.43)	1.49	0.136	1.91	(0.86)
	Endorsement of EU cooperation	0.01	(0.01)	1.91	0.056	1.01	(0.01)
	Civic knowledge	0.00	(0.00)	-3.58	0.000	1.00	(0.00)
	Gender (girls vs. boys)	0.03	(0.10)	0.31	0.754	1.03	(0.10)
	Immigration status	-0.05	(0.07)	-0.73	0.464	0.95	(0.06)
	Expected further education	-0.01	(0.06)	-0.19	0.852	0.99	(0.06)
	SES	-0.12	(0.05)	-2.31	0.021	0.89	(0.05)

Table S6. Results from the binary logistic regression using "European cooperation to guarantee high levels of employment" and selected predictors.

Educational system	Variable	Coefficients	(SE)	Wald Statistic	p	Odds Ratio	(SE)
Belgium (Flemish)	(Intercept)	0.75	(1.03)	0.73	0.467	2.16	(2.31)
	Civic knowledge	0.01	(0.00)	4.00	0.000	1.01	(0.00)
	Gender (girls vs. boys)	-0.18	(0.19)	-0.95	0.342	0.83	(0.16)
	Immigration status	-0.42	(0.23)	-1.81	0.070	0.66	(0.15)
	Expected further education	0.15	(0.10)	1.55	0.121	1.17	(0.12)
	SES	-0.11	(0.11)	-0.98	0.329	0.89	(0.10)
Bulgaria	(Intercept)	-3.70	(1.92)	-1.93	0.054	0.03	(0.09)
	Civic knowledge	0.01	(0.00)	8.31	0.000	1.01	(0.00)
	Gender (girls vs. boys)	-0.07	(0.22)	-0.33	0.742	0.93	(0.21)
	Immigration status	0.94	(0.61)	1.53	0.126	2.55	(1.36)
	Expected further education	-0.20	(0.11)	-1.91	0.057	0.82	(0.09)
	SES	0.16	(0.10)	1.51	0.131	1.17	(0.12)
Croatia	(Intercept)	-3.66	(1.93)	-1.89	0.058	0.03	(0.08)
	Civic knowledge	0.01	(0.00)	2.67	0.008	1.01	(0.00)
	Gender (girls vs. boys)	0.67	(0.53)	1.25	0.212	1.95	(1.01)
	Immigration status	0.95	(0.51)	1.87	0.062	2.60	(1.18)
	Expected further education	-0.03	(0.31)	-0.10	0.918	0.97	(0.30)
	SES	-0.20	(0.27)	-0.77	0.440	0.81	(0.22)
Denmark	(Intercept)	-1.53	(1.02)	-1.50	0.133	0.22	(0.24)
	Civic knowledge	0.01	(0.00)	4.92	0.000	1.01	(0.00)
	Gender (girls vs. boys)	-0.31	(0.17)	-1.81	0.070	0.73	(0.12)
	Immigration status	0.24	(0.20)	1.20	0.229	1.28	(0.25)
	Expected further education	0.14	(0.15)	0.88	0.380	1.14	(0.18)
	SES	-0.05	(0.10)	-0.52	0.603	0.95	(0.09)
Estonia	(Intercept)	-3.37	(0.99)	-3.39	0.001	0.03	(0.03)
	Civic knowledge	0.01	(0.00)	7.67	0.000	1.01	(0.00)
	Gender (girls vs. boys)	-0.52	(0.23)	-2.26	0.024	0.59	(0.14)
	Immigration status	0.31	(0.22)	1.39	0.166	1.36	(0.31)
	Expected further education	0.16	(0.08)	2.10	0.035	1.18	(0.09)
	SES	-0.04	(0.10)	-0.38	0.707	0.96	(0.10)
Finland	(Intercept)	-0.71	(1.27)	-0.56	0.575	0.49	(0.90)
	Civic knowledge	0.01	(0.00)	5.74	0.000	1.01	(0.00)
	Gender (girls vs. boys)	0.07	(0.24)	0.32	0.751	1.08	(0.26)
	Immigration status	-0.36	(0.38)	-0.96	0.335	0.70	(0.23)
	Expected further education	0.11	(0.15)	0.74	0.458	1.12	(0.16)
	SES	0.16	(0.12)	1.38	0.168	1.18	(0.14)
Germany, North-Rhine Westphalia	(Intercept)	1.50	(1.56)	0.96	0.335	4.52	(9.67)
	Civic knowledge	0.00	(0.00)	1.33	0.184	1.00	(0.00)
	Gender (girls vs. boys)	-0.08	(0.22)	-0.37	0.711	0.92	(0.21)
	Immigration status	0.03	(0.28)	0.09	0.926	1.03	(0.29)
	Expected further education	-0.07	(0.16)	-0.47	0.637	0.93	(0.15)

Educational system	Variable	Coefficients	(SE)	Wald Statistic	p	Odds Ratio	(SE)
	SES	0.31	(0.17)	1.88	0.060	1.36	(0.23)
Italy	(Intercept)	-2.59	(0.62)	-4.15	0.000	0.08	(0.05)
	Civic knowledge	0.01	(0.00)	8.42	0.000	1.01	(0.00)
	Gender (girls vs. boys)	0.05	(0.14)	0.40	0.688	1.06	(0.14)
	Immigration status	0.18	(0.14)	1.29	0.197	1.20	(0.17)
	Expected further education	0.03	(0.09)	0.31	0.758	1.03	(0.09)
	SES	0.01	(0.09)	0.14	0.888	1.01	(0.09)
Latvia	(Intercept)	-1.63	(0.84)	-1.95	0.052	0.20	(0.16)
	Civic knowledge	0.01	(0.00)	4.70	0.000	1.01	(0.00)
	Gender (girls vs. boys)	-0.25	(0.19)	-1.34	0.181	0.78	(0.14)
	Immigration status	0.47	(0.25)	1.87	0.061	1.60	(0.41)
	Expected further education	0.09	(0.09)	0.94	0.346	1.09	(0.10)
	SES	0.04	(0.12)	0.36	0.719	1.04	(0.12)
Lithuania	(Intercept)	-1.02	(1.11)	-0.92	0.359	0.37	(0.44)
	Civic knowledge	0.01	(0.00)	4.47	0.000	1.01	(0.00)
	Gender (girls vs. boys)	0.09	(0.16)	0.53	0.595	1.09	(0.18)
	Immigration status	0.08	(0.30)	0.28	0.777	1.09	(0.31)
	Expected further education	-0.05	(0.14)	-0.39	0.699	0.95	(0.13)
	SES	0.00	(0.10)	-0.01	0.993	1.00	(0.10)
Malta	(Intercept)	-1.77	(0.76)	-2.33	0.020	0.17	(0.13)
	Civic knowledge	0.01	(0.00)	10.77	0.000	1.01	(0.00)
	Gender (girls vs. boys)	0.19	(0.20)	0.96	0.339	1.21	(0.24)
	Immigration status	0.09	(0.22)	0.42	0.677	1.10	(0.24)
	Expected further education	0.03	(0.09)	0.28	0.781	1.03	(0.10)
	SES	0.03	(0.10)	0.32	0.751	1.03	(0.11)
Netherlands	(Intercept)	-1.26	(0.72)	-1.74	0.083	0.29	(0.20)
	Civic knowledge	0.01	(0.00)	6.59	0.000	1.01	(0.00)
	Gender (girls vs. boys)	-0.01	(0.15)	-0.08	0.938	0.99	(0.15)
	Immigration status	-0.03	(0.22)	-0.15	0.880	0.97	(0.22)
	Expected further education	-0.01	(0.08)	-0.09	0.926	0.99	(0.08)
	SES	-0.01	(0.10)	-0.06	0.950	0.99	(0.10)
Norway	(Intercept)	-0.78	(0.51)	-1.52	0.128	0.46	(0.24)
	Civic knowledge	0.01	(0.00)	7.83	0.000	1.01	(0.00)
	Gender (girls vs. boys)	0.20	(0.13)	1.52	0.129	1.22	(0.16)
	Immigration status	0.01	(0.13)	0.08	0.933	1.01	(0.13)
	Expected further education	0.09	(0.08)	1.06	0.288	1.09	(0.09)
	SES	0.10	(0.06)	1.65	0.098	1.11	(0.07)
Slovenia	(Intercept)	-3.23	(1.15)	-2.81	0.005	0.04	(0.06)
	Civic knowledge	0.01	(0.00)	7.14	0.000	1.01	(0.00)
	Gender (girls vs. boys)	-0.01	(0.31)	-0.03	0.977	0.99	(0.30)
	Immigration status	-0.23	(0.22)	-1.04	0.299	0.80	(0.17)
	Expected further education	0.24	(0.16)	1.46	0.144	1.27	(0.20)
	SES	-0.12	(0.15)	-0.81	0.419	0.89	(0.13)

Educational system	Variable	Coefficients	(SE)	Wald Statistic	p	Odds Ratio	(SE)
Sweden	(Intercept)	0.94	(0.82)	1.15	0.252	2.61	(2.29)
	Civic knowledge	0.00	(0.00)	2.41	0.016	1.00	(0.00)
	Gender (girls vs. boys)	-0.30	(0.22)	-1.35	0.178	0.74	(0.16)
	Immigration status	-0.31	(0.21)	-1.49	0.136	0.73	(0.15)
	Expected further education	0.30	(0.10)	3.09	0.002	1.35	(0.13)
	SES	-0.06	(0.13)	-0.48	0.634	0.94	(0.12)

Table S7. Results from the binary logistic regression using "European countries should cooperate to strengthen their economies" and selected predictors.

Educational system	Variable	Coefficients	(SE)	Wald Statistic	p	Odds Ratio	(SE)
Belgium (Flemish)	(Intercept)	-0.29	(0.80)	-0.37	0.712	0.75	(0.62)
	Civic knowledge	0.01	(0.00)	4.12	0.000	1.01	(0.00)
	Gender (girls vs. boys)	-0.07	(0.18)	-0.40	0.693	0.93	(0.17)
	Immigration status	-0.16	(0.17)	-0.97	0.332	0.85	(0.14)
	Expected further education	0.15	(0.10)	1.56	0.119	1.16	(0.11)
	SES	-0.27	(0.13)	-2.10	0.036	0.77	(0.10)
Bulgaria	(Intercept)	-5.62	(1.66)	-3.38	0.001	0.00	(0.01)
	Civic knowledge	0.01	(0.00)	7.46	0.000	1.01	(0.00)
	Gender (girls vs. boys)	0.42	(0.17)	2.44	0.015	1.52	(0.26)
	Immigration status	1.26	(0.52)	2.40	0.016	3.51	(1.79)
	Expected further education	-0.07	(0.09)	-0.76	0.447	0.93	(0.08)
	SES	-0.04	(0.11)	-0.35	0.723	0.96	(0.11)
Croatia	(Intercept)	-2.34	(1.51)	-1.55	0.121	0.10	(0.16)
	Civic knowledge	0.01	(0.00)	3.98	0.000	1.01	(0.00)
	Gender (girls vs. boys)	0.14	(0.27)	0.52	0.606	1.15	(0.31)
	Immigration status	0.07	(0.38)	0.18	0.854	1.07	(0.42)
	Expected further education	0.22	(0.19)	1.16	0.246	1.25	(0.24)
	SES	-0.35	(0.17)	-2.05	0.041	0.71	(0.12)
Denmark	(Intercept)	1.67	(0.77)	2.17	0.030	5.40	(4.23)
	Civic knowledge	0.00	(0.00)	2.95	0.003	1.00	(0.00)
	Gender (girls vs. boys)	-0.08	(0.17)	-0.46	0.646	0.93	(0.16)
	Immigration status	-0.30	(0.22)	-1.37	0.169	0.74	(0.16)
	Expected further education	0.08	(0.10)	0.88	0.377	1.09	(0.10)
	SES	0.10	(0.07)	1.52	0.129	1.11	(0.08)
Estonia	(Intercept)	-4.10	(1.29)	-3.19	0.001	0.02	(0.02)
	Civic knowledge	0.01	(0.00)	6.70	0.000	1.01	(0.00)
	Gender (girls vs. boys)	-0.18	(0.22)	-0.78	0.433	0.84	(0.19)
	Immigration status	0.38	(0.28)	1.36	0.173	1.46	(0.43)
	Expected further education	-0.10	(0.11)	-0.84	0.400	0.91	(0.10)
	SES	-0.10	(0.13)	-0.77	0.443	0.91	(0.12)
Finland	(Intercept)	-0.54	(1.05)	-0.51	0.608	0.59	(0.62)
	Civic knowledge	0.01	(0.00)	4.05	0.000	1.01	(0.00)
	Gender (girls vs. boys)	0.10	(0.23)	0.43	0.664	1.11	(0.25)
	Immigration status	0.12	(0.31)	0.38	0.701	1.13	(0.36)
	Expected further education	0.17	(0.11)	1.50	0.134	1.18	(0.13)
	SES	-0.03	(0.12)	-0.20	0.838	0.98	(0.12)
Germany, North-Rhine Westphalia	(Intercept)	0.80	(0.98)	0.81	0.416	2.31	(2.52)
	Civic knowledge	0.01	(0.00)	2.85	0.004	1.01	(0.00)
	Gender (girls vs. boys)	0.27	(0.19)	1.40	0.160	1.31	(0.26)
	Immigration status	-0.20	(0.18)	-1.08	0.281	0.82	(0.15)
	Expected further education	-0.32	(0.09)	-3.70	0.000	0.72	(0.06)

Educational system	Variable	Coefficients	(SE)	Wald Statistic	p	Odds Ratio	(SE)
	SES	0.32	(0.17)	1.87	0.061	1.38	(0.25)
Italy	(Intercept)	-1.75	(0.71)	-2.47	0.014	0.17	(0.13)
	Civic knowledge	0.01	(0.00)	8.78	0.000	1.01	(0.00)
	Gender (girls vs. boys)	0.04	(0.20)	0.21	0.836	1.04	(0.21)
	Immigration status	-0.11	(0.18)	-0.60	0.547	0.90	(0.16)
	Expected further education	-0.04	(0.10)	-0.38	0.703	0.96	(0.09)
	SES	0.02	(0.09)	0.20	0.838	1.02	(0.09)
Latvia	(Intercept)	-0.54	(1.10)	-0.49	0.624	0.59	(0.62)
	Civic knowledge	0.01	(0.00)	6.00	0.000	1.01	(0.00)
	Gender (girls vs. boys)	0.11	(0.17)	0.64	0.522	1.11	(0.19)
	Immigration status	-0.11	(0.35)	-0.33	0.743	0.89	(0.32)
	Expected further education	0.01	(0.12)	0.06	0.952	1.01	(0.13)
	SES	0.00	(0.08)	-0.04	0.970	1.00	(0.08)
Lithuania	(Intercept)	-1.61	(2.51)	-0.64	0.521	0.20	(1.52)
	Civic knowledge	0.01	(0.00)	6.13	0.000	1.01	(0.00)
	Gender (girls vs. boys)	0.33	(0.24)	1.35	0.176	1.39	(0.33)
	Immigration status	-0.43	(0.81)	-0.53	0.598	0.65	(0.41)
	Expected further education	0.41	(0.12)	3.39	0.001	1.50	(0.18)
	SES	-0.15	(0.16)	-0.91	0.362	0.86	(0.14)
Malta	(Intercept)	-2.73	(0.61)	-4.46	0.000	0.07	(0.04)
	(Intercept)	0.01	(0.00)	11.47	0.000	1.01	(0.00)
	Civic knowledge	0.27	(0.18)	1.55	0.121	1.31	(0.23)
	Gender (girls vs. boys)	0.08	(0.17)	0.49	0.628	1.08	(0.18)
	Immigration status	0.14	(0.07)	2.06	0.039	1.15	(0.08)
	Expected further education	0.08	(0.09)	0.93	0.355	1.09	(0.10)
Netherlands	(Intercept)	-0.13	(0.99)	-0.13	0.894	0.88	(0.83)
	Civic knowledge	0.01	(0.00)	4.94	0.000	1.01	(0.00)
	Gender (girls vs. boys)	-0.12	(0.19)	-0.63	0.531	0.89	(0.17)
	Immigration status	-0.25	(0.20)	-1.27	0.202	0.78	(0.16)
	Expected further education	-0.01	(0.09)	-0.08	0.940	0.99	(0.09)
	SES	0.16	(0.10)	1.58	0.115	1.18	(0.12)
Norway	(Intercept)	2.63	(0.58)	4.53	0.000	13.92	(8.32)
	Civic knowledge	0.00	(0.00)	1.75	0.081	1.00	(0.00)
	Gender (girls vs. boys)	0.08	(0.11)	0.74	0.461	1.08	(0.12)
	Immigration status	-0.43	(0.13)	-3.32	0.001	0.65	(0.08)
	Expected further education	0.06	(0.06)	1.14	0.255	1.07	(0.06)
	SES	-0.05	(0.06)	-0.82	0.415	0.95	(0.06)
Slovenia	(Intercept)	-4.22	(0.75)	-5.64	0.000	0.01	(0.01)
	Civic knowledge	0.01	(0.00)	8.25	0.000	1.01	(0.00)
	Gender (girls vs. boys)	0.17	(0.17)	1.03	0.304	1.19	(0.20)
	Immigration status	0.30	(0.16)	1.83	0.068	1.34	(0.22)
	Expected further education	0.23	(0.12)	1.94	0.052	1.26	(0.15)
	SES	-0.23	(0.11)	-2.18	0.029	0.80	(0.08)

Educational system	Variable	Coefficients	(SE)	Wald Statistic	p	Odds Ratio	(SE)
Sweden	(Intercept)	1.10	(0.72)	1.52	0.130	3.06	(2.30)
	Civic knowledge	0.00	(0.00)	1.89	0.059	1.00	(0.00)
	Gender (girls vs. boys)	0.13	(0.17)	0.79	0.428	1.14	(0.19)
	Immigration status	-0.12	(0.19)	-0.64	0.525	0.89	(0.16)
	Expected further education	0.17	(0.10)	1.65	0.098	1.18	(0.12)
	SES	-0.02	(0.16)	-0.13	0.893	0.98	(0.16)
